# A powerful and versatile colocalization test

**DOI:** 10.1371/journal.pcbi.1007778

**Published:** 2020-04-10

**Authors:** Yangqing Deng, Wei Pan

**Affiliations:** Division of Biostatistics, University of Minnesota, Minneapolis, Minnesota, United States of America; University of Virginia, UNITED STATES

## Abstract

Transcriptome-wide association studies (TWAS and PrediXcan) have been increasingly applied to detect associations between genetically predicted gene expressions and GWAS traits, which may suggest, however do not completely determine, causal genes for GWAS traits, due to the likely violation of their imposed strong assumptions for causal inference. Testing colocalization moves it closer to establishing causal relationships: if a GWAS trait and a gene’s expression share the same associated SNP, it may suggest a regulatory (and thus putative causal) role of the SNP mediated through the gene on the GWAS trait. Accordingly, it is of interest to develop and apply various colocalization testing approaches. The existing approaches may each have some severe limitations. For instance, some methods test the null hypothesis that there is colocalization, which is not ideal because often the null hypothesis cannot be rejected simply due to limited statistical power (with too small sample sizes). Some other methods arbitrarily restrict the maximum number of causal SNPs in a locus, which may lead to loss of power in the presence of wide-spread allelic heterogeneity. Importantly, most methods cannot be applied to either GWAS/eQTL summary statistics or cases with more than two possibly correlated traits. Here we present a simple and general approach based on conditional analysis of a locus on multiple traits, overcoming the above and other shortcomings of the existing methods. We demonstrate that, compared with other methods, our new method can be applied to a wider range of scenarios and often perform better. We showcase its applications to both simulated and real data, including a large-scale Alzheimer’s disease GWAS summary dataset and a gene expression dataset, and a large-scale blood lipid GWAS summary association dataset. An R package “jointsum” implementing the proposed method is publicly available at github.

## Author summary

In the post-GWAS era, colocalization testing has been playing an increasingly important role in inferring causal genetic variants and causal genes from GWAS trait-associated loci. However, colocalization testing is challenging. We first discuss some severe limitations of the existing methods, thus motivating our development of a general and powerful approach. We use extensive simulations to demonstrate the advantages of our approach over other existing methods. To further demonstrate the performance differences, we apply our and other methods (when possible) for colocalization analyses of multiple correlated GWAS traits and that of a GWAS trait and gene expression.

## Introduction

Genome-wide association studies (GWAS) have identified many loci associated with complex traits or diseases, but causal relationships cannot be established, leading to lack of mechanistic understandings [1,2]. Testing colocalization of a GWAS trait and a gene’s expression trait (i.e. that the same variant is causal to both traits) can help gain insight into the mechanism[3]. For example, if it can be established that a causal variant for a GWAS trait and that for a gene’s expression (i.e. expression quantitative trait locus, eQTL) are the same, then it may suggest a regulatory role of the causal SNP on gene expression in the pathway to the GWAS trait [4,5], which can be also regarded as vertical pleiotropy (i.e., the SNP affects both traits but the effect on the GWAS trait is mediated by the gene expression). Some integrative methods, such as transcriptome-wide association studies (TWAS) [6], PrediXcan [7], summary statistics-based Mendelian randomization (SMR) [8] and some related methods [9,10], have been proposed recently to detect association between (the genetically regulated component of) a gene’s expression (or another molecular trait) and a GWAS trait. Under their causal inference frameworks, these methods may suggest, but cannot fully determine, a colocalization, because the imposed assumptions may be violated such as due to linkage disequilibrium (LD) [11] and horizontal pleiotropy (i.e., the SNP affects both traits but the effect on the GWAS trait is not mediated by the gene expression) [12,13]. Besides detecting colocalization of possibly causal SNPs for a GWAS trait and a molecular trait like gene expression, it is also of interest for colocalization analysis of multiple complex traits or diseases, which for example may be helpful for understanding the shared biological pathways for multiple diseases and thus for drug repurposing and new therapeutic development. Studies have found that some diseases seem to have commonly associated genetic variants [14,15], which by themselves cannot determine colocalization either; when a variant is associated with multiple traits, it could be due to distinct causal SNPs that are in linkage disequilibrium (LD) [16], which however can be distinguished through joint/conditional modeling of multiple SNPs as in fine mapping [17,18,19]. This critical difference between marginal and conditional associations also highlights the difference between the existing pleiotropy testing [20,21] and the proposed colocalization testing on multiple traits.

To test colocalization in a formal way, there have been extensive efforts in developing and applying various approaches. However, they all have some severe limitations as briefly discussed below. First, the popular coloc [22] and HEIDI [8] can be both considered as proportional approaches. A possible downside is that their null hypothesis specifies proportional effect sizes as a result of colocalization, which will not be rejected if the sample size is too small or the significance level is too stringent. Hence, smaller studies are more likely to conclude with colocalization, which is undesirable. Second, many (e.g. coloc, HEIDI and JLIM [23]) assume the presence of no more than one causal SNP in a locus, inconsistent with the recently discovered widespread allelic heterogeneity [24,25,26]; when the assumption is violated, they may suffer from substantial power loss. Third, although eCAVIAR [27] does not impose such an assumption, it requires specifying an upper bound on the number of causal SNPs in a locus, typically at 6, to be computationally feasible. Such an assumption when violated may lead to power loss as shown for real data [26]. Fourth, some (e.g. JLIM) require the availability of individual-level genotypic and gene expression data, which may not be practical for large-scale GWAS with only summary statistics available. Fifth, at least with their current implementations, most of the existing methods, with few exceptions [28,29], cannot handle more than two traits with dependent/overlapping samples with only summary statistics from genome-wide association studies for all the traits, which is important for multi-trait analysis to identify pleiotropy.

We develop a simple and general approach overcoming the above shortcomings of the existing methods. We call this approach the conditional method, due to the central role of conditional modeling in fine mapping; in addition, more general than most existing methods, our approach applies to multiple traits with only summary statistics based on one or more possibly dependent/overlapping samples. We demonstrate that, in certain scenarios, the new method has power advantages over the existing methods using simulated data. Also, to show our method's flexibility as well as its ability to detect significant loci, we compare different methods using the large-scale GWAS lipid data [30] with four traits and summary statistics only, and to the largest AD GWAS by IGAP [31] with GWAS summary statistics only and an individual-level gene expression dataset from ADNI [32].

## Methods

### Existing methods

We will compare our method with some major representatives for the existing methods, namely JLIM, coloc, coloc.abf, HEIDI and eCAVIAR, the details of which can be found in the S1 Text. To summarize, JLIM needs individual level data for the second trait; JLIM, coloc, coloc.abf and HEIDI mainly aim at the scenario with at most one causal SNP for each trait in a locus; eCAVIAR is more robust when allelic heterogeneity (AH) exists (i.e. there are multiple causal SNPs for a trait), but it still requires giving a (usually small) maximum number of causal SNPs (to limit the computational burden).

### Testing colocalization with conditional analysis

Suppose we are interested in *p* traits and *q* SNPs, and we want to test whether at least one of the SNPs is causal for all the traits. We first assume the availability of individual-level data, and will discuss its extension to GWAS summary data at the end. The *k*th SNP and the *j*th trait in the individual level data are **X**_*k*_ = (*X*_1*k*_…*X*_*nk*_)′ and **Y**_*j*_ = (*Y*_1*j*_…*Y*_*nj*_)′, where *n* is the sample size. With the individual level data, we can easily build a conditional model for each trait as

$$Y_{j}=Xb_{j}+e_{j},$$ (1)

where **X** = (**X**_1_…**X**_*q*_), **b**_*j*_ = (*b*_*j*1_…*b*_*jq*_)′, and **e**_*j*_ = (*e*_1*j*_…*e*_*nj*_)′; **e**_*j*_ is assumed to be independently normal with mean 0. The null and alternative hypotheses for our colocalization test are

$$H_{0}:\;no\;k\;satisfies\;b_{1k}\neq 0,b_{2k}\neq 0,\ldots ,b_{pk}\neq 0;$$

$$H_{1}:\;at\;least\;one\;k\;satisfies\;b_{1k}\neq 0,b_{2k}\neq 0,\ldots ,b_{pk}\neq 0.$$

Suppose the estimated coefficients and standard errors are ${\hat{b}}_{jk}$ and $se({\hat{b}}_{jk})$, where *k* = 1,…,*q* and *j* = 1,…,*p*. We can also get the p-value for ${\hat{b}}_{jk}$, denoted by *P*_*jk*_. To test *H*_0_, we can apply the principles of the Intersection-Union Test (IUT) and the Union-Intersection Test (UIT) [33,34]. We divide the null hypothesis into simpler sub-hypotheses like

$$H_{0}=\underset{k}{⋂}H_{0k}=\underset{k}{⋂}\underset{j}{⋃}H_{0jk},$$

$$H_{1k}:\;b_{1k}\neq 0,b_{2k}\neq 0,\ldots ,b_{pk}\neq 0,$$

$$H_{0k}={H_{1k}}^{c},$$

$$H_{0jk}:\;b_{jk}=0.$$

*P*_*jk*_ can be considered as the test statistic for testing *H*_0*jk*_. By IUT, max_*j*_(*P*_*jk*_) is a valid test statistic for *H*_0*k*_ = ⋃_*j*_*H*_0*jk*_. If max_*j*_(*P*_*jk*_) is small enough for SNP *k*, it suggests colocalization at this position/SNP since all of its *P*_*jk*_'s are small (i.e., its effects on all traits are significant). By UIT, min_*k*_[max_*j*_(*P*_*jk*_)] is a valid test statistic for *H*_0_ = ⋂_*k*_*H*_0*k*_. If min_*k*_[max_*j*_(*P*_*jk*_)] is small enough, it suggests there is colocalization since at least one SNP's max_*j*_(*P*_*jk*_) is small enough. Thus we can simply define our test statistic for *H*_0_ as

$$P^{*}=\underset{k}{\min}[\underset{j}{\max}\left(P_{jk}\right)].$$

Suppose the given nominal statistical significance level is *α*. The colocalization test can be carried out by rejecting *H*_0_ if P*<*α*, which means for at least one SNP, its effects on all of the traits are nonzero (p-value < *α*). However, this approach involves multiple testing, which will lead to inflated type I errors. Hence, we propose using the Bonferroni correction: we reject *H*_0_ if and only if P*<*α*/*q*. In this way, the type I error rate is guaranteed to be controlled.

If we have only GWAS summary statistics, instead of individual-level data, we can also perform the colocalization test. Denote the estimated marginal effect size of the *k*th SNP on the *j*th trait and its variance by ${\hat{\beta }}_{jk}$ and $\hat{var}({\hat{\beta }}_{jk})$, respectively. Suppose we also have some reference panel to estimate the LD among the SNPs. We can use some well-known method, e.g. described in [18], to estimate the coefficients and standard errors in each conditional model (1). Now after obtaining our estimates ${\hat{b}}_{jk}$ and $se({\hat{b}}_{jk})$, we can get the p-values and conduct the above colocalization test.

### The conditional method with monte carlo approximation

Since the Bonferroni correction may be quite conservative, we propose another way to calculate the p-value, called the conditional method with Monte Carlo approximation (**CMC**), to test colocalization, which is expected to be less conservative. For convenience, we denote the previously described conditional method with Bonferroni correction by **CB**. Suppose **Z** = (*Z*_*jk*_) is the Z-statistic matrix with $Z_{jk}={\hat{b}}_{jk}/se({\hat{b}}_{jk})$ for trait *j* and SNP *k*. Assume that the covariance/correlation matrices are constant across the rows and columns, denoted as *Cov*(***Z***_*j*_.) = *corr*(***Z***_*j*_.) = ***R***_1_, and *Cov*(***Z***_.*k*_) = *corr*(***Z***_.*k*_) = ***R***_2_. ***R***_1_ and ***R***_2_ are usually estimated by and regarded as the correlations among the SNPs and among the traits respectively. Our test statistic is

$$T_{cond}=\underset{k}{\max}(\underset{j}{\min}\left|Z_{jk}\right|).$$

Suppose that the observed test statistic is *t*. The p-value is

$$P(\underset{k}{\max}\left(\underset{j}{\min}\left|Z_{jk}\right|\right)>t)=1-P(\underset{k}{\max}\left(\underset{j}{\min}\left|Z_{jk}\right|\right)\leq t).$$

If we know the mean of *Z*_*jk*_'s under the null, we can calculate *P*(max_*k*_(min_*j*_|*Z*_*jk*_|)≤*t*) by using multivariate normal tail probabilities or Monte Carlo simulations. The challenge here is that the means of *Z*_*jk*_'s are unknown under *H*_0_, which is a composite null hypothesis. For instance, it is possible that the first SNP has some effect on the first trait but not the second trait, which means *Z*_11_ has a nonzero mean while *Z*_21_ has a zero mean. It is also possible that the first SNP does not have any effect at all, meaning that *Z*_11_ and *Z*_21_ both have a zero mean. In different situations, the distribution of max_*k*_(min_*j*_|*Z*_*jk*_|) can vary a lot, which we will show numerically in the simulation section. What we know is for each SNP *k*, at most (*p*−1) of the *Z*_*jk*_'s have a nonzero mean under the null and at least one has a zero mean. A conservative way to get around is to assume all *Z*_11_,…,*Z*_1*q*_ have a zero mean and use

$$P(\underset{k}{\max}\left(\underset{j}{\min}\left|Z_{jk}\right|\right)>t)\leq P(\underset{k}{\max}\left(\left|Z_{1k}\right|\right)>t)=1-P(|Z_{11}|<t,\ldots ,|Z_{1q}|<t),$$

where *P*(|*Z*_11_|<*t*,…,|*Z*_1*q*_|<*t*) is now a calculable tail probability. The problem of this approach is that it is quite conservative, due to its use of the above inequality.

We propose to use the current data to roughly estimate which of the *Z*_*jk*_'s have nonzero means. For each SNP *k*, we compare its |*Z*_*jk*_|'s with |Φ^−1^(*θ*/2)|, where Φ is the probability function of the standard normal distribution and *θ* is a tuning parameter, usually chosen as 0.05 or 0.1. We will discuss more about it later. If |*Z*_j*k_| is greater than |Φ^−1^(*θ*/2)|, we assume under the null, SNP *k* is associated with trait j*; otherwise SNP *k* has no effect on trait j*. Since each SNP must have no effect on at least one trait under the null (no colocalization), we always assume the effect corresponding to the smallest |*Z*_*jk*_| to be 0 regardless of how large this |*Z*_*jk*_| is. Let △ be the set of all the trait-SNP pairs (*j*,*k*) whose corresponding effects are assumed to be nonzero. Then we assume for each *k*,

$$\underset{j}{\min}\left(|Z_{jk}|\right)=\underset{j:(j,k)\notin △}{\min}\left(|Z_{jk}|\right),$$

which means |*Z*_*jk*_|'s with nonzero means are always larger than the other |*Z*_*jk*_|'s under the null. If none of the |*Z*_*jk*_|'s is greater than |Φ^−1^(*θ*/2)|, we assume SNP *k* has no effect on any trait under the null, so *Z*_*jk*_'s all have a zero mean. In this way, we can estimate

$$P(\underset{k}{\max}\left(\underset{j}{\min}\left|Z_{jk}\right|\right)\leq t)=P(\underset{j:\;(j,1)\notin △}{\min}\left|Z_{j1}\right|<t,\ldots ,\underset{j:\;(j,q)\notin △}{\min}\left|Z_{jq}\right|<t).$$

Now all of the *Z*_*jk*_'s on the right-hand side have a zero mean, and their covariance matrix can be easily constructed with ***R***_1_ and ***R***_2_, because

$$Cov((Z_{j1},\ldots ,Z_{jq})\prime )=R_{1},$$

$$Cov((Z_{1k},\ldots ,Z_{pk})\prime )=R_{2},$$

$$Cov(Z_{jk},Z_{j\prime k\prime })=0\;(j\neq j\prime ,k\neq k\prime ).$$

We can break the whole probability down into tail probabilities that can be calculated by the "pmvnorm" function, as shown in the appendix, but the number of tail probabilities is usually too big (e.g. 3^*q*^) due to the limitation of the function, which makes it computationally infeasible for large *q*'s. A better way is to use a Monte Carlo approximation. For each iteration *b*, we can simulate *Z*_*jk*_^(*b*)^ 's that satisfy *Z*_*jk*_^(*b*)^∉△ from a multivariate normal distribution with zero mean and then calculate $t^{\left(b\right)}=\max_{k}\min_{j:{Z_{\mathrm{jk}}}^{\left(b\right)}\notin △}\left|{Z_{jk}}^{\left(b\right)}\right|$. Then the p-value is simply

$$\frac{1}{B}\sum_{b=1}^{B}I\{t^{\left(b\right)}>t\}.$$

Note that it is equivalent to simulate all of the *Z*_*jk*_^(*b*)^ 's from MVN(***μ***,**Σ**) and calculate *t*^(*b*)^ = max_*k*_ min_*j*_|*Z*_*jk*_^(*b*)^|, where **Σ** = ***R***_2_⊗***R***_1_ and ***μ*** = (*μ*_11_,…,*μ*_1*q*_,*μ*_21_,…,*μ*_2*q*_,…,*μ*_*p*1_,…,*μ*_*pq*_)′. Now *μ*_*jk*_ = 0 if *Z*_*jk*_∉△ and *μ*_*jk*_ = Inf otherwise, for defining *μ*_*jk*_ = Inf results in $P(\max_{k}\left(\min_{j}\left|Z_{jk}\right|\right)\leq t)=P(\min_{j:Z_{j1}\notin △}\left|Z_{j1}\right|<t,\ldots ,\min_{j:Z_{\mathrm{jq}}\notin △}\left|Z_{jq}\right|<t)$. In practice, we can use a large value like 999 for *μ*_*jk*_ (j, k: *Z*_*jk*_∈△). For convenience, we denote this method by **CMC**. The choice of *B* depends on the significance level *α*. In the simulations where we aim at *α* = 0.05,*B* = 10^4^ is usually more than enough. For the real data application where *α* = 5×10^−8^ is often considered, we can try *B* = 10^4^ first. If the p-value of one locus turns out to be less than 0.005, we can try *B* = 10^6^. If the new p-value turns out to be less than 5×10^−5^, we can use *B* = 10^9^ to get a final p-value for that locus. We will follow this proposal in the next sections unless otherwise specified.

### Model averaging

The previously described process uses |*Z*_*jk*_|'s to determine the null distribution and then uses Monte Carlo simulations to estimate a p-value. Nevertheless, its performance may be affected if the null distribution is not optimally determined (i.e. the assumed △ is far off from the truth). Following the model averaging idea from [35], we can use different △'s to get different p-values, and then take the weighted average to get a final p-value. The process to get different △'s is:

(1) Order |*Z*_*jk*_|'s. Suppose (*j*_1_,*k*_1_) has the largest |*Z*_*jk*_|, (*j*_2_,*k*_2_) has the second largest |*Z*_*jk*_|, etc.

(2) △_0_ is *ϕ*, the empty set, meaning that all of the means are zero.

(3) Examine each trait-SNP pair (*j*_1_,*k*_1_), (*j*_2_,*k*_2_), etc. Suppose currently the biggest △ we have is △_*t*_ and the trait-SNP pair we are looking at is (*j*_*l*_,*k*_*l*_).

Check whether (*j*_*l*_,*k*_*l*_) satisfies both

- $\left|Z_{j_{l}k_{l}}\right|$ is larger than a cutoff *ξ*.
- After adding (*j*_*l*_,*k*_*l*_) into △_*t*_, it still satisfies the null hypothesis (i.e. the new △ does not contain all (*j*,*k*_*l*_)'s with the same *k*_*l*_).

If (*j*_*l*_,*k*_*l*_) satisfies these two conditions, then △_*t*+1_ = △_*t*_∪{(*j*_*l*_,*k*_*l*_)}. If not, move to the next pair (*j*_*l*+1_,*k*_*l*+1_) and check the conditions again. Continue this process until one of the following conditions is met:

- All of the |*Z*_*jk*_|'s that are larger than *ξ* have been examined.
- We already have △_0_,△_1_,…,△_*u*_, where *u* is a pre-specified number.

For each △_*t*_, we can use the approach in the previous subsection to get a p-value *P*_*t*_. We can also get a weight, *w*_*t*_, for this p-value using the AICc value [36] as in [35], details of which are provided in S1 Text. Then the final p-value for testing colocalization is ∑_*t*_*w*_*t*_*P*_*t*_/∑_*t*_*w*_*t*_. For short, we call this method **MA-CMC**, or simply **MA**. If *ξ* is too small, it can lead to inflated type I errors. Our recommended value for *ξ* is 1.64. To save computing time, we recommend using a moderate *u* (e.g. 10 by default). Note that when we use the above process to get different △'s, if the number of SNPs is large (e.g. 100), instead of adding one SNP at a time like described in step (3), it may be beneficial to add multiple SNPs at a time. For example, if we add one SNP at a time, the largest size of △ (or number of nonzero effects) is *u*. If we add *s* SNPs at a time, the largest number of nonzero effects that can be considered is *su*.

### Selecting the tuning parameters

As mentioned before, CMC depends on a tuning parameter *θ*. When *θ* is small, CMC will be less conservative, but it can have inflated type I errors as well. When *θ* is large, CMC will be more conservative with loss of power. As a result, we recommend choosing a *θ* that is not too large or too small (e.g. 0.1). One way to interpret *θ* is that if the mean of *Z*_*jk*_ is zero, the probability of getting |*Z*_*jk*_|>|Φ^−1^(*θ*/2)| will be *θ*. More extensive simulation studies and discussions on choosing *θ* are provided in the S1 Text, based on which we decide to use *θ* = 0.1 by default. MA involves two more tuning parameters *s* and *u*. We recommend choosing *u* = 10 and modifying *s* according to the number of SNPs in each locus (e.g. *s* = 1 for *q*≤20; *s* = 2 for 20<*q*≤50; *s* = 3 for *q*>50). This choice gives us a fairly reasonable *su*, which means the largest number of nonzero effects that can be considered under the null. When *su* is fixed, choosing a larger *u* and a smaller *s* means adding more models. This usually leads to higher power but also larger type I errors, partly because the more models we add, the more likely one or more of them have significant results with none-negligible weights. Based on our experience and to keep it simple, we suggest using *u* = 10 by default, though cautions have to be taken (e.g. with possible sensitivity analyses).

### Package availability

The coloc method uses the "coloc.test" function (for hypothesis testing) and “coloc.abf” (for drawing ROC curves) from the R "coloc" package. The HEIDI method was implemented by the original authors in the "smr" software available at http://cnsgenomics.com/software/smr/#SMR&HEIDIanalysis, while the eCAVIAR method is available at http://genetics.cs.ucla.edu/caviar/download.html. The JLIM method was originally implemented as the "jlim.test" function in the "jlimR" package, which requires input files in specific formats (e.g. need to include unnecessary SNP information); we re-implemented JLIM in our own R function, which only needs the p-values, a reference panel and permuted genotypes as input. This function for JLIM and the functions for CB, CMC and MA are included in the R package "jointsum", publicly available at https://github.com/yangq001/conditional.

## Results

### Simulations

#### Possible null distributions

**Fig 1:**
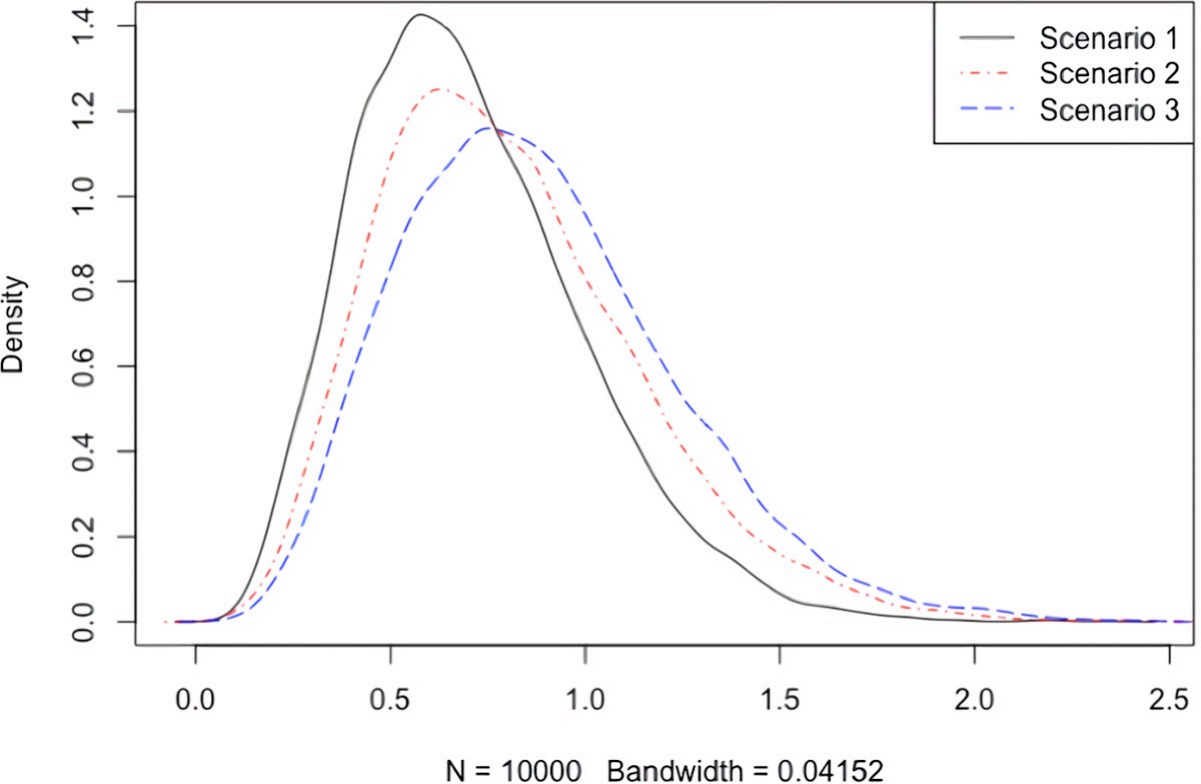
Density curves of *T*_conditional_ in 3 different null scenarios based on 10000 replications. Scenario 1: All *Z*_*jk*_'s are centered at 0. Scenario 2: *Z*_11_, *Z*_21_, *Z*_12_, *Z*_22_ are centered at 1. Scenario 3: *Z*_11_, *Z*_21_, *Z*_12_, *Z*_22_, *Z*_13_, *Z*_23_, *Z*_14_, *Z*_24_ are centered at 1. In all scenarios, *Z*_*jk*_'s are independent and have variance 1.

First, we use a simple example to demonstrate that the null distribution of the test statistic of the conditional method largely depends on the unknown truth of which effects are nonzero under the composite null hypothesis. Suppose we have 5 independent SNPs and 3 independent traits. Fig 1 shows the different densities of the test statistic *T*_conditional_ under different null scenarios. In the first scenario, no SNP has any effect on any trait. In the second scenario, the first two SNPs have nonzero effects on the first two traits. In the third scenario, the first four SNPs have nonzero effects on the first two traits. Since no SNP has nonzero effects on all of the three traits, the null hypothesis holds (i.e., there is no colocalization). As shown in Fig 1, the null distribution of the test statistic changes dramatically with different scenarios. The 0.95-quantiles are 1.24, 1.42 and 1.51 respectively. If the truth is scenario 3, but we use the null distribution in scenario 1 to generate test statistics, then it will lead to inflated type I errors; it is not hard to imagine the scenarios of using an inappropriate null distribution would lead to substantial power loss too. This confirms the importance of estimating the nonzero components as we did for CMC and MA.

Next, we did some simulation studies to compare the performances of different methods (CB, CMC, MA, JLIM, etc.).

#### Independent samples

We simulated two traits for *q* SNPs in a small region in chromosome 19, making sure none of the correlations' absolute values were greater than 0.9. For the first trait, we selected the first 2000 subjects from the 4136 subjects in the Lung Health Study (LHS) data, and then used a linear model **Y** = **Xβ**+**ε** to obtain the trait, where **β** = (*β*_1_…*β*_*q*_), and **ε** was independently normally distributed with mean 0 and variance 1. *β*_*k*_ was nonzero when the *k*th SNP was causal for the first trait. For the second trait, we used the rest of the subjects and a similar model Y* = X*β*+ε*. Note that **X** and X* represented the same set of SNPs but for different samples, so the two traits, based on two non-overlapping samples, were independent. We regressed **Y** and Y* separately on each SNP using the corresponding subjects to get the summary statistics of marginal associations for each trait-SNP pair. The individual level genotype data were also used as our reference panels.

We compared the conditional method with various approaches (i.e., without Bonferroni adjustment, with Bonferroni adjustment, CMC, MA) along with JLIM, coloc and HEIDI, using the suggested tuning parameters for the methods (e.g. the threshold for neighborhood in JLIM was $\sqrt{0.8}$). We used 500 permutations for JLIM. In addition to conducting these regional tests, we also obtained the results of testing whether a certain SNP *k* is causal for both traits. Simply reject the null hypothesis if both p-values of this SNP are smaller than *α*. The p-values can be from the marginal models or the conditional models. For our simulations, we only included the case looking at the first SNP using the conditional p-values. To distinguish testing one SNP and testing a region in conditional analysis, we indicate the former by "1st" in the tables.

First, we looked at the type I errors of JLIM, conditional methods, CMC and MA. In this scenario, the rejection rates of coloc and HEIDI were their empirical power. As shown in Table 1, the conditional method with Bonferroni’s adjustment for multiple testing was conservative, as expected, while CMC and MA were less conservative. The type I error rate of CMC went up as *θ* went down. As for JLIM, it had inflated type I errors in the last situation because it only looked at marginal effects. Due to its correlation with other SNPs, a non-causal SNP might turn out to have the most significant marginal association. In this case, SNPs 1 and 5 were causal for the first trait, but the correlation between these two SNPs and SNP 4 made SNP 4 the most marginally significant. As a result, JLIM tended to falsely conclude with colocalization, yielding larger type I errors. We also discovered that JLIM had very low rejection rates when the causal SNP for trait 2 was not SNP 1, which is due to JLIM's definition of neighborhoods. Detailed explanations can be found in S1 Text.

**Table 1:** Rejection rates (type I errors for JLIM and conditional, power for coloc and HEIDI). *q* = 13. 1000 iterations. *α* = 0.05. The correlation is -0.69 between SNP 1 and SNP 3, 0.68 between SNP 1 and SNP 4, 0.02 between SNP 1 and SNP 5, -0.35 between SNP 4 and SNP 5. Different subjects for two traits. For CMC, *B* = 10^4^. For MA, *B* = 10^3^, *u* = 10 and *s* = 1.

Causal locations for trait 1 (size)	Causal locations for trait 2 (size)	JLIM	Conditional				coloc	HEIDI**
			1^st^ SNP	CB (w/o adj.)	CMC*	MA		
1 (0.3)	None	0.047	0.054	0.005 (0.079)	0.031 (0.021)[0.015]	0.020	0.056	0.194 (0.006)
1 (0.3)	3 (0.3)	0	0.054	0.007 (0.117)	0.039 (0.025) [0.018]	0.025	0.964	0.076 (0.059)
1 (0.3)	4 (0.3)	0	0.054	0.007 (0.121)	0.039 (0.029)[0.020]	0.030	0.974	0.040 (0.038)
1 (0.3)	4 (-0.3)	0	0.054	0.007 (0.121)	0.051 (0.029) [0.025]	0.031	0.970	0.042 (0.040)
1 (0.2), 5 (-0.3)	4 (0.3)	0.393	0.049	0.009 (0.145)	0.048 (0.028)[0.022]	0.030	0.932	0.090 (0.085)

* The tuning parameter *θ* was chosen as 0.05 (0.1) [0.2].

** When there was no causal location for trait 2, HEIDI sometimes output NA (after detecting the violation of its assumptions). We thus recorded two ratios A (B). A: #rejected/#non-NA results. (B): #rejected/#iterations.

Next, we looked at the power of different methods. Since the conditional method without adjustment had already been demonstrated to have inflated type I errors, we did not include this approach anymore. For convenience, the conditional method will refer to the conditional method with Bonferroni correction from this point unless otherwise specified. Besides, a smaller *θ* was shown to make CMC less conservative but it would also possibly lead to inflated type I errors, so we chose *θ* = 0.1, a medium value, from this point on. More simulation results and discussions on the choice of *θ* are provided in S1 Text. As Table 2 shows, when both causal locations were SNP 1, the conditional method testing SNP 1 had lower power than JLIM, which was probably due to estimating all those parameters in the conditional model. However, sometimes JLIM had very low power when there were more than one causal SNP for a trait. For example, when SNP 1 was causal for trait 1, and both SNP 1 and SNP 4 were causal for trait 2, but the effect of SNP 4 was stronger, JLIM tended to take SNP 4 as the only causal location for trait 2. As a result, it concluded that there was no colocalization, which led to loss of power. In contrast, the conditional method was able to distinguish multiple causal SNPs and thus maintaining its power in this case. Using CMC had much higher power than using the Bonferroni adjustment. Here, MA performed similarly to CMC, but we will show later that sometimes the former was more powerful.

**Fig 2:**
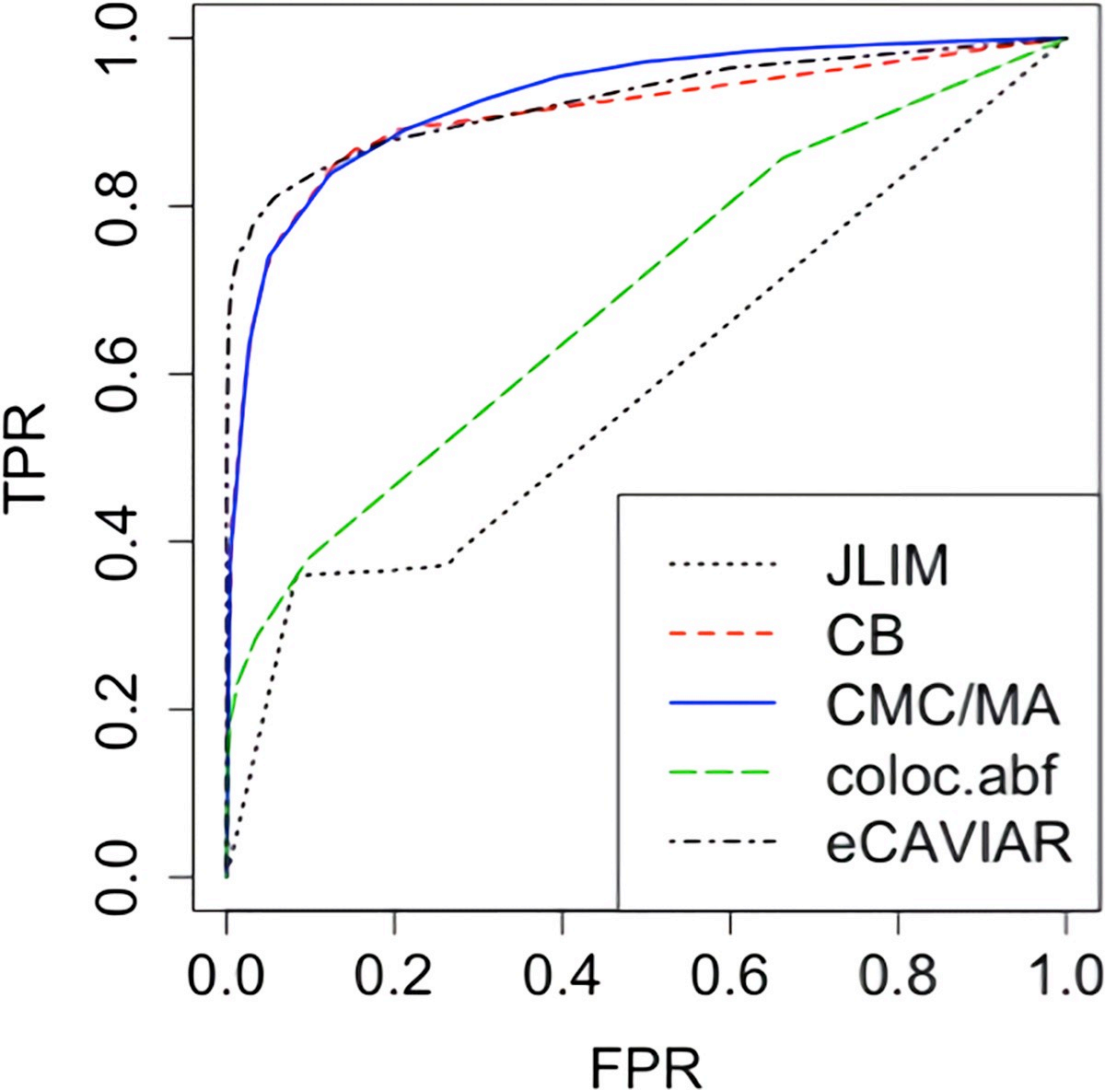
ROC curve of JLIM, CB, CMC, eCAVIAR and coloc (Bayesian), combining all the scenarios in Table 1 and Table 2 (12000 samples in total).

In addition, we compared the receiver operating characteristic (ROC) curves of JLIM, CB, CMC and coloc, as well as eCAVIAR (with the maximum number of causal SNPs set to 3). This time we used the more recent and popular Bayesian version (coloc.abf) of coloc, which gives the posterior probability of having one common causal variant [29,37]. We combined the scenarios in Table 1 and Table 2 to calculate the true positive rates (TPR) and false positive rates (FPR). Due to the close performance of CMC and MA, only the curve for CMC was plotted for better visualization. As shown in Fig 2, JLIM did not perform well at all since its assumption (at most 1 causal SNP) was often violated. The assumption of eCAVIAR (at most 3 causal SNPs) was sometimes violated too. In the last two scenarios in Table 2 with more causal SNPs, eCAVIAR had lower power because it failed to detect all the causal SNPs and thus was less likely to establish colocalization. As a result, when all the scenarios were combined, eCAVIAR did not have much advantage. Meanwhile, CB and CMC were quite robust and did better than the Bayesian coloc (using default settings) in these scenarios.

**Table 2:** Rejection rates (power for JLIM and conditional methods, type I errors for coloc and HEIDI). *q* = 13. 1000 iterations. Different subjects for two traits. For CMC, *B* = 10^3^. For MA, *B* = 10^3^, *u* = 10 and *s* = 1.

Causal locations for trait 1 (size)	Causal locations for trait 2 (size)	JLIM	conditional				Coloc	HEIDI
			1^st^ SNP	CB	CMC	MA		
1 (0.2)	1 (0.2)	0.977	0.798	0.391	0.640	0.636	0.041	0.105 (0.048)
1 (0.7)	1 (0.2)	0.986	0.911	0.646	0.823	0.820	0.045	0.230 (0.105)
1 (0.2)	1 (0.2), 4 (0.4)	0.011	0.798	0.392	0.611	0.611	0.488	0.020 (0.020)
1 (0.2), 4(0.2)	1 (0.2)	0.514	0.798	0.395	0.613	0.623	0.262	0.171 (0.078)
1 (0.2), 4(0.4)	1 (0.2)	0.009	0.798	0.397	0.612	0.617	0.548	0.327 (0.210)
1 (0.2)	1 (0.2), 2 (0.3), 3 (0.3), 4 (0.4)	0.004	0.798	0.393	0.567	0.565	0.971	0.595 (0.549)
1 (0.2), 2 (0.3), 3 (0.4), 4 (0.5)	1 (0.2)	0.004	0.798	0.399	0.575	0.568	0.992	0.776 (0.524)

Note that JLIM's ROC curve seemed to have an uncommon shape in Fig 1. The main reason is that JLIM had many p-values of 0 or 1. We have already shown that sometimes JLIM had zero rejection rates in some scenarios in Table 1. Most of the p-values in those cases were 1. Likewise, when the alternative was true, because of the way of constructing the test statistic, the p-values of JLIM tended to be exactly 0 once it successfully picked up the right causal SNP. We tried increasing the number of permutations, but it did not really change the result.

#### Dependent samples

In the above scenarios, we assumed the data for the two traits came from different subjects. We also looked at some other situations where the two traits were correlated from the same subjects. This time we only used the 2000 subjects previously used for trait 1. The models for two traits were **Y** = **Xβ**+**ε** and Y* = Xβ*+ε*. For subject *i*, the correlation between **ε**_*i*_ and ε*_i_ was 0.5.

As shown in Table 3, the results were similar to the previous ones. The conditional method with Bonferroni adjustment was conservative, and CMC was less conservative. MA had higher power than CMC. JLIM had better performance in some cases, but in others it could have inflated type I errors or very low power because of its problematic assumption of at most one causal SNP for each trait (and that this causal SNP should be marginally the most significant). The conditional method did not have this issue at all.

**Table 3:** Rejection rates. *q* = 13. 1000 iterations. *α* = 0.05. Same subjects for two traits. For CMC, *B* = 10^3^. Colocalization = No: type I errors for JLIM and conditional, power for coloc. Colocalization = Yes: power for JLIM and conditional, type I errors for coloc. For MA, *B* = 10^3^, *u* = 10 and *s* = 1.

Colocalization	Causal locations for trait 1 (size)	Causal locations for trait 2 (size)	JLIM	Conditional				coloc	HEIDI
				1^st^ SNP	CB	CMC	MA		
No	1 (0.3)	None	0.042	0.057	0.002	0.030	0.032	0.029	0.164 (0.027)
	1 (0.3)	4 (0.3)	0	0.057	0.004	0.035	0.041	0.954	0.012 (0.011)
	1 (0.2), 5 (-0.3)	4 (0.3)	0.395	0.040	0.004	0.029	0.028	0.849	0.040 (0.034)
Yes	1 (0.2)	1 (0.2)	0.980	0.815	0.439	0.621	0.680	0.001	0.017 (0.014)
	1 (0.2)	1 (0.2), 4 (0.4)	0.013	0.815	0.439	0.584	0.655	0.292	0.006 (0.006)

We did some other simulations that were closer to real scenarios, involving larger numbers of SNPs and various effect sizes. The genotypes were obtained from all the subjects (around 4000) in the LHS data. We used the Lipid data from the Global Lipids Genetics Consortium GWAS study [30] to get some loci on chromosome 1 and set up the linear models to simulate 2 traits, LDL and HDL. To determine the loci, we expanded from the marginally significant SNPs for LDL in a similar way to what was done by [38]. We also made sure none of the correlations were greater than 0.95. Then we used the Lipid data with the 1000G data as reference to build linear models $\tilde{Y}=\tilde{X}\tilde{\beta }+\tilde{\varepsilon }$ and ${\tilde{Y}}^{*}=\tilde{X}{\tilde{\beta }}^{*}+{\tilde{\varepsilon }}^{*}$ where $\tilde{Y}$ and ${\tilde{Y}}^{*}$ here stand for LDL and HDL, $\tilde{X}$ is the genotypes of the subjects used for the Lipid data. The models we used to simulate two traits were **Y** = **Xβ**+**ε** and $Y^{*}=X\beta^{*}+\varepsilon^{*}$ where **X** is the genotypes from the LHS data. **β** and β* were obtained by shrinking smaller effects (<*τ*) in $\tilde{\beta }$ and ${\tilde{\beta }}^{*}$ to 0 and multiplying larger effects (≥*τ*) by 2. Then we applied the methods in the same way as previous. The "true" effect sizes in each region can be found in S1 Text.

The first part of Table 4 shows the results for 2 regions (A1 and A2) with no colocalization. Both JLIM and CMC were able to control type I errors, while the conditional method with Bonferroni adjustment was much more conservative. Coloc did not reject its null hypothesis, probably because its assumption of only one causal SNP for each trait was violated. The second part of the table contains several examples of loci with colocalization (regions B1-B6). CMC and MA worked well and showed higher power than JLIM and CB in most cases. JLIM often had very low power when applied to these loci, because it only focused on the most significant SNP for each trait, while the SNP(s) with colocalization usually did not have the largest marginal effect size. In the meantime, coloc had very high rejection rates because there were many causal SNPs and the proportionality assumption did not hold. These results are consistent with what we had in Table 2 (rows 3, 5, 6 and 7).

**Table 4:** Rejection rates. 1000 iterations. *α* = 0.05. Same subjects for two traits. For CMC, *B* = 10^3^. *τ* = 0.2. Regions A1-A2: without colocalization (type I errors for JLIM and conditional, power for coloc). Regions B1-B6: with colocalization (power for JLIM and conditional, type I errors for coloc). For MA, *B* = 10^3^, *u* = 10. *s* = 3 for loci with more than 50 SNPs, *s* = 2 otherwise.

Region	# SNPs (*)	JLIM	Conditional				coloc
			1^st^ SNP	CB	CMC	MA	
A1	43 (2/0/0)	0.039	0.011	0	0.014	0.026	0.027
A2	58 (17/2/0)	0.054	0.010	0.010	0.030	0.046	0.047
B1	28 (9/4/1)	0	0.018	0.189	0.276	0.288	1
B2	53 (7/18/2)	0	0.005	0.429	0.542	0.581	1
B3	62 (12/6/2)	0.724	0.011	0.546	0.721	0.739	1
B4	43 (18/10/5)	0	0.009	0.154	0.238	0.281	1
B5	43 (9/7/6)	0.179	0.016	0.683	0.859	0.877	1

(*) Numbers of SNPs that are causal for trait 1 / trait 2 / both traits.

### Lipid Data: colocalization of two GWAS traits

We looked at the Lipid data from the Global Lipids Genetics Consortium GWAS study [30], containing summary statistics for traits LDL, HDL, TG and TC. The 1000 Genomes Project data [39] were used as the reference panel. To compare the performance of different methods, we applied the conditional methods and coloc to test colocalization of only two traits, LDL and HDL, in different regions, and we also tested each SNP in the marginal models and each lead SNP in the conditional models for colocalization. First, we focused on several chromosomes that had multiple SNPs associated with both LDL and HDL (marginal p-value < 5e-8). Then we looked at the SNPs that were also present in the 1000G data (with 503 subjects) on those chromosomes. Some of the SNPs were removed because they did not appear in the 1000G data. Instead of using sliding windows, we defined the loci associated with LDL following the procedure by [38]. JLIM could not be applied to this scenario because individual level data was not available for either trait. The marginal analysis looking at one SNP at a time examined each SNP on the chromosomes of interest, while the other methods only looked at the defined loci associated with LDL.

According to Table 5, the conditional method with Bonferroni correction did not detect any significant loci while CMC found many, proving that CMC can be much less conservative. MA's result was close to CMC's. Coloc's result was different, likely because its null hypothesis was the opposite of the other methods' while the chosen cutoff was the same; this might lead to many false discoveries.

**Table 5:** Numbers of SNPs and loci with colocalization. LDL and HDL only. For the methods that test each locus, *α* = 0.05/*#*loci. For the marginal method that tests each SNP separately, the numbers are the numbers of significant SNPs, and *α* = 0.05/(*#*SNPs in "Marginal"). The numbers in parentheses were obtained using the cut-off α = 5E-8. For coloc, the numbers of loci with colocalization are the ones that were not rejected for its null hypothesis under *α*.

Chr	Marginal		Regional						
	# SNPs	# Significant SNPs	# Loci	# SNPs	# Loci with Colocalization				# Significant Lead SNPs*
					CB	CMC	MA	Coloc	
1	181458	19 (17)	26	134	0 (0)	9 (4)	9 (4)	16 (21)	6/9
2	209518	77 (55)	33	230	0 (0)	16 (3)	16 (3)	13 (22)	5/13
11	124669	129 (114)	26	239	0 (0)	23 (19)	24 (19)	8 (21)	20/23
12	116372	10 (7)	2	17	0 (0)	1 (0)	1 (0)	0 (2)	0/1
16	67080	32 (29)	5	40	0 (0)	3 (0)	3 (0)	3 (5)	1/3
19	33311	21 (18)	21	81	0 (0)	7 (4)	7 (4)	10 (13)	2/7
20	58830	1 (1)	6	45	0 (0)	1 (0)	1 (0)	1 (2)	1/1

* Testing lead SNPs with *α* = 0.05/(*#*SNPs in marginal). Lead SNPs were defined as the ones with the smallest p-values for LDL / smallest p-value sums for LDL + HDL. These p-values were obtained from the conditional models.

### Lipid Data: colocalization of more than two traits

Using the conditional method with Bonferroni correction or CMC, we are also able to test colocalization of more than 2 traits, while the other methods we discussed can only be applied to 2 traits. We tested colocalization for LDL, HDL and TG with the same SNPs and loci as in the previous section. As shown in Table 6, CMC detected more colocalization loci than the conditional method with Bonferroni correction. The results of testing loci and testing lead SNPs were relatively consistent.

**Table 6:** Numbers of loci with colocalization. LDL, HDL and TG. For the methods testing each locus, *α* = 0.05/#loci. For the marginal method that tests each SNP separately, the numbers are the numbers of significant SNPs, and *α* = 0.05/(*#*SNPs in "Marginal"). For testing lead SNPs, *α* = 0.05/(*#*SNPs in "Regional").

Chr	Marginal		Regional					
	# SNPs	# Significant SNPs	# Loci	# SNPs	# Loci with Colocalization			# Significant Lead SNPs*
					CB	CMC	MA	
1	181434	6	26	134	6	6	7	4/4
2	209518	70	33	230	11	11	12	5/9
11	124669	125	26	239	22	23	23	20/21
12	116372	0	2	17	1	1	1	0/1
16	67080	28	5	40	1	1	1	0/0
19	33311	16	21	81	5	5	6	5/3
20	58830	0	6	45	1	1	1	0/1

* Lead SNPs were defined as the ones with the smallest p-values for LDL / smallest p-value sums for LDL + HDL + TG. These p-values were obtained from the conditional models.

In addition, we tested colocalization of all four traits, LDL, HDL, TG and TC with the new methods. This time we used the same gap length and window size to obtain large non-overlapping windows, and then applied the new methods to each window. We also applied the marginal analysis looking at one SNP at a time to see whether a SNP was associated with all the traits (all p-values < *α*). Note that the reference data (1000G) only have 503 subjects while the window size is as large as 100. The joint models we build may be quite inaccurate. Hence, we applied the Sum-MI method in [40], which uses the reference panel and marginal summary statistics to build joint models more effectively. Then we used the summary statistics (joint effects and their covariance matrix) from these joint models for the conditional method.

As Table 7 shows, in the presence of four traits, the conditional methods were able to detect multiple significant loci with summary statistics. MA had the most significant results.

**Table 7:** Numbers of significant SNPs and windows. Window size = 100. Test colocalization of LDL, HDL, TG and TC. For MA, *u* = 10 and *s* = 5.

Chr	Marginal		Regional			
	# SNPs	# Significant SNPs (*α* = 0.05/*#*SNPs)	# Windows	# Significant windows (*α* = 0.05/*#*windows)		
				CB	CMC	MA
2	209518	48	2095	1	4	6
12	116372	0	1163	0	0	3
16	67080	27	670	0	0	1
19	33311	16	333	1	1	3

## IGAP and ADNI Data: colocalization of AD and gene expression

**Fig 3:**
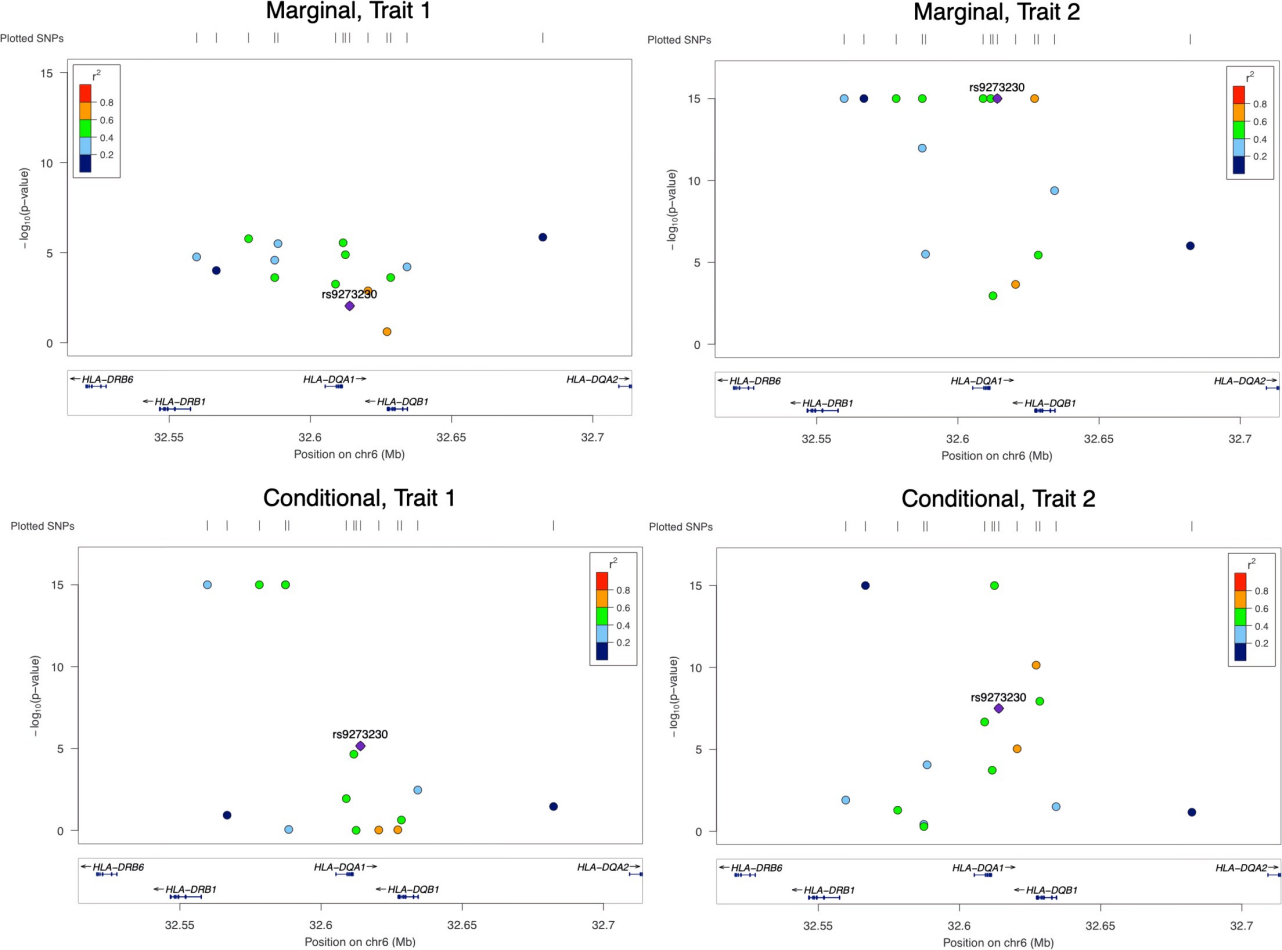
One locus associated with schizophrenia on chromosome 6. JLIM did not detect colocalization but the conditional method's result was more significant. Top: LocusZoom plots of 15 SNPs' p-values in marginal analysis. Bottom: LocusZoom plots of the same SNPs' p-values in conditional analysis. Smaller p-values are truncated at 1e-15.

We applied the methods to the largest AD GWAS data by IGAP (International Genomics of Alzheimer's Project) [31], along with the ADNI (Alzheimer's Disease Neuroimaging Initiative) data [32] to detect colocalization of AD and gene expressions. CMC and MA's results were more significant than CB's. JLIM and the conditional methods had different results, mostly because JLIM looked at the marginal effects while the conditional methods looked at the joint effects. Fig 3 is a LocusZoom plot [41] that shows the difference between JLIM and the conditional method. For this locus, JLIM did not detect colocalization (its p-value was almost 1), while the conditional method did with a looser threshold (p-value = 8e-7). Note that JLIM only looked at the marginal effects while the conditional method examined the conditional ones. None of the SNPs were marginally significant enough for both traits, so JLIM did not conclude colocalization. However, in the conditional analysis, the p-values of some SNPs for trait 1 became much smaller. As a result, the conditional method gave a much smaller p-value. More details and other examples are provided in S1 Text.

## Discussion

We have presented a new method to test colocalization with conditional analysis, which plays a central role in fine mapping to distinguish causal SNPs from marginally associated ones due to the latter’s LD with the former. The proposed conditional method offers some distinct advantages over other existing methods in many scenarios. First of all, unlike coloc and HEIDI, the conditional method's null hypothesis is that there is no colocalization, which seems more natural and desirable for the purpose of detecting colocalization. Besides, compared to methods like JLIM, the conditional method does not put any restriction on the number of causal SNPs in a locus. It also considers conditional effects of SNPs in a locus, rather than marginal effects, which could be quite different. As shown in the simulation study and discussed in the literature [17,18], when multiple SNPs are in LD, the marginally most significant SNP may not be one of the causal ones, in which case JLIM may draw a wrong conclusion due to its relying on the marginally most significant SNP. JLIM may also ignore a SNP's causal effect when another SNP's effect is stronger. However, the conditional method can handle these cases with ease. The difference between marginal and conditional analyses is also shown in our real data examples. In addition, some methods require individual level data for at least one trait, while the conditional method can be easily applied to GWAS summary statistics with a reference panel. Finally, among the methods we have compared, the conditional method is the only one that is currently available for dealing with more than two traits from either independent or overlapping samples.

We have developed two new methods called CMC and MA-CMC (or simply MA), which are less conservative than the Bonferroni correction, in conditional analysis of multiple SNPs in a candidate locus. The performances of CMC and MA have been shown to improve over many existing methods both in simulations and real data applications. A technical challenge in colocalization testing with conditional analysis is the nature of the composite null hypothesis: the null distribution of the test statistic (under *H*_0_) is unknown (with some unknown nuisance parameters); specifically, under *H*_0_, although we have the asymptotic distribution of (*Z*_*jk*_) as a matrix normal variate, but for any given *j* and *k*, the mean of *Z*_*jk*_ is unknown that may not equal to 0. In CMC, we have proposed a simple method to estimate these non-zero means/components; it can be made more conservative with the choice of a larger tuning parameter value (closer to 1) to estimate an upper bound of the true p-value, yielding a type I error rate under the nominal significance level with possible loss of power; we proposed a default value that seems to be working well across a wide range of simulations. However, CMC may also suffer from power loss when the estimation of non-zero components is too difficult to be good. To deal with this problem, we proposed to use MA, which looks at multiple estimates/choices of non-zero components and takes the weighted average of different results. As long as one of those choices is close to the truth, it can benefit MA's power, making it even less conservative than CMC. One limitation of MA is that it not only requires a cut off value as CMC does, but also needs a pre-specified number of effects to be added each time as well as the total number of models. Furthermore, MA requires more computing time. In addition, we tried the harmonic mean p-value (HMP) by [42] for the conditional method to replace the Bonferroni correction, but the improvement was very limited (so we did not include the results). In the future, it will be worthwhile to further explore other options to yield less conservative (and thus more powerful) colocalization tests in conditional analysis. One possible way to make the new method more efficient is to apply a sequential Monte Carlo method [43]. Finally, we would like to mention that, as for any challenging problem in practice, it might be more informative to apply multiple approaches, instead of a single one, to reach a more robust conclusion. Our proposed methods as hypothesis testing can be applied first to examine if there is any colocalization. If so, a Bayesian approach may be used to identify the SNPs that are likely to be causal for multiple traits. The first step focuses on the global question of the presence/absence of colocalization, while the second step aims at identifying specific causal SNPs, which is more challenging and may require larger samples.

## Supporting information

S1 Text A supplementary file contains a brief review on some main representatives of the existing methods, plots of effect sizes for Table 4, results for IGAP and ADNI data and some other technical discussions. (DOCX)

## References

[1] Guo H, Fortune MD, Burren OS, Schofield E, Todd JA, Wallace C. Integration of disease association and eQTL data using a Bayesian colocalisation approach highlights six candidate causal genes in immune-mediated diseases. Hum Mol Genet. 2015 6 15;24(12):3305–13. 10.1093/hmg/ddv077 25743184 PMC4498151

[2] Gallagher MD, Chen-Plotkin AS. The Post-GWAS Era: From Association to Function. *Am J Hum Genet*. 2018;102(5):717–730. 10.1016/j.ajhg.2018.04.002 29727686 PMC5986732

[3] Cannon ME, Mohlke KL. Deciphering the Emerging Complexities of Molecular Mechanisms at GWAS Loci. *Am J Hum Genet*. 2018;103(5):637–653. 10.1016/j.ajhg.2018.10.001 30388398 PMC6218604

[4] He X, Fuller CK, Song Y, Meng Q, Zhang B, Yang X, et al Sherlock: detecting gene-disease associations by matching patterns of expression QTL and GWAS. Am J Hum Genet. 2013 5 2;92(5):667–80. 10.1016/j.ajhg.2013.03.022 23643380 PMC3644637

[5] Ongen H, Brown AA, Delaneau O, Panousis NI, Nica AC, GTEx Consortium, et al Estimating the causal tissues for complex traits and diseases. *Nat Genet*. 2017;49:1676–83. 10.1038/ng.3981 29058715

[6] Gusev A, Ko A, Shi H, Bhatia G, Chung W, Penninx BWJH, et al Integrative approaches for large-scale transcriptome-wide association studies. *Nat Genet*. 2016 3;48(3):245–52. 10.1038/ng.3506 26854917 PMC4767558

[7] Gamazon ER, Wheeler HE, Shah KP, Mozaffari SV, Aquino-Michaels K, Carroll RJ, et al A gene-based association method for mapping traits using reference transcriptome data. Nat Genet. 2015 9;47(9):1091–8. 10.1038/ng.3367 26258848 PMC4552594

[8] Zhu Z, Zhang F, Hu H, Bakshi A, Robinson MR, Powell JE, et al Integration of summary data from GWAS and eQTL studies predicts complex trait gene targets. *Nature Genetics*, 2016 5; 48(5), 481–7. 10.1038/ng.3538 27019110

[9] Xu Z, Wu C, Wei P, Pan W. A Powerful Framework for Integrating eQTL and GWAS Summary Data. *Genetics*. 2017;207(3):893–902. 10.1534/genetics.117.300270 28893853 PMC5676241

[10] Su YR, Di C, Bien S, Huang L, Dong X, Abecasis G, et al A Mixed-Effects Model for Powerful Association Tests in Integrative Functional Genomics. Am J Hum Genet. 2018 5 3;102(5):904–919. 10.1016/j.ajhg.2018.03.019 29727690 PMC5986723

[11] Mancuso N, Kichaev G, Shi H, Freund M, Gusev A, Pasaniuc B. Probabilistic fine-mapping of transcriptome-wide association studies. bioRxiv, 2017, 10.1101/236869 PMC6619422 30926970

[12] Wang T, Moon JY, Wu Y, Amos CI, Hung RJ, Tardon A, et al Pleiotropy of genetic variants on obesity and smoking phenotypes: Results from the Oncoarray Project of The International Lung Cancer Consortium. PLoS One. 2017 9 28;12(9):e0185660 10.1371/journal.pone.0185660 28957450 PMC5619832

[13] Verbanck M, Chen CY, Neale B, Do R. Detection of widespread horizontal pleiotropy in causal relationships inferred from Mendelian randomization between complex traits and diseases. *Nat Genet*. 2018;50(5):693–698. 10.1038/s41588-018-0099-7 29686387 PMC6083837

[14] Cotsapas C, Voight BF, Rossin E, Lage K, Neale BM, Wallace C, et al Pervasive Sharing of Genetic Effects in Autoimmune Disease. *PLoS Genet*. 2011;7(8):e1002254 10.1371/journal.pgen.1002254 21852963 PMC3154137

[15] Sha Q, Wang Z, Zhang X, Zhang S. A clustering linear combination approach to jointly analyze multiple phenotypes for GWAS. *Bioinformatics*, 2019 4 15;35(8):1373–1379. 10.1093/bioinformatics/bty810 30239574 PMC6477981

[16] Fortune MD, Guo H, Burren O, Schofield E, Walker NM, Ban M, et al Statistical Colocalization of Genetic Risk Variants for Related Autoimmune Diseases in the Context of Common Controls. *Nat Genet*. 2015;47(7):839–846. 10.1038/ng.3330 26053495 PMC4754941

[17] Yang J, Ferreira T, Morris AP, Medland SE, Genetic Investigation of ANthropometric Traits (GIANT) Consortium, DIAbetes Genetics Replication And Meta-analysis (DIAGRAM) Consortium, et al Conditional and joint multiple-SNP analysis of GWAS summary statistics identifies additional variants influencing complex traits. Nat Genet. 2012 3 18;44(4):369–75, S1-3. 10.1038/ng.2213 22426310 PMC3593158

[18] Deng Y, Pan W. Conditional analysis of multiple quantitative traits based on marginal GWAS summary statistics. *Genet Epidemiol*. 2017;41(5):427–436. 10.1002/gepi.22046 28464407 PMC5536980

[19] Schaid DJ, Chen W, Larson NB. From genome-wide associations to candidate causal variants by statistical fine-mapping. Nat Rev Genet. 2018 8;19(8):491–504. 10.1038/s41576-018-0016-z 29844615 PMC6050137

[20] Schaid DJ, Tong X, Larrabee B, Kennedy RB, Poland GA, Sinnwell JP. Statistical Methods for Testing Genetic Pleiotropy. Genetics. 2016 10;204(2):483–497. 10.1534/genetics.116.189308 27527515 PMC5068841

[21] Deng Y, Pan W. Testing Genetic Pleiotropy with GWAS Summary Statistics for Marginal and Conditional Analyses. *Genetics*. 2017;207(4): 1285–1299. 10.1534/genetics.117.300347 28971959 PMC5714448

[22] Wallace C, Rotival M, Cooper JD, Rice CM, Yang JH, McNeill M, et al Statistical colocalization of monocyte gene expression and genetic risk variants for type 1 diabetes. Hum Mol Genet. 2012 6 15;21(12):2815–24. 10.1093/hmg/dds098 22403184 PMC3363338

[23] Chun S, Casparino A, Patsopoulos NA, Croteau-Chonka DC, Raby BA, De Jager PL, et al Limited statistical evidence for shared genetic effects of eQTLs and autoimmune-disease-associated loci in three major immune-cell types. Nat Genet. 2017 4;49(4):600–605. 10.1038/ng.3795 28218759 PMC5374036

[24] Jansen R, Hottenga JJ, Nivard MG, Abdellaoui A, Laport B, de Geus EJ, et al Conditional eQTL analysis reveals allelic heterogeneity of gene expression. Hum Mol Genet. 2017 4 15;26(8):1444–1451. 10.1093/hmg/ddx043 28165122 PMC6075455

[25] Hormozdiari F, Zhu A, Kichaev G, Ju CJ, Segrè AV, Joo JWJ, et al Widespread Allelic Heterogeneity in Complex Traits. Am J Hum Genet. 2017 5 4;100(5):789–802. 10.1016/j.ajhg.2017.04.005 28475861 PMC5420356

[26] Deng Y, Pan W. Significance Testing for Allelic Heterogeneity. *Genetics*. 2018;210: 25–32. 10.1534/genetics.118.301111 29959179 PMC6116971

[27] Hormozdiari F, van de Bunt M, Segrè AV, Li X, Joo JWJ, Bilow M, et al Colocalization of GWAS and eQTL Signals Detects Target Genes. Am J Hum Genet. 2016 12 1;99(6):1245–1260. 10.1016/j.ajhg.2016.10.003 27866706 PMC5142122

[28] Wen X, Pique-Regi R, Luca F. Integrating molecular QTL data into genome-wide genetic association analysis: Probabilistic assessment of enrichment and colocalization. *PLoS Genet*. 2017 3 13(3): e1006646 10.1371/journal.pgen.1006646 28278150 PMC5363995

[29] Giambartolomei C, Zhenli Liu J, Zhang W, Hauberg M, Shi H, Boocock J, et al A Bayesian framework for multiple trait colocalization from summary association statistics. Bioinformatics. 2018 8 1;34(15):2538–2545. 10.1093/bioinformatics/bty147 29579179 PMC6061859

[30] Willer CJ, Schmidt EM, Sengupta S, Peloso GM, Gustafsson S, Kanoni S, et al Discovery and refinement of loci associated with lipid levels. *Nat Genet*. 2013;45(11):1274–1283. 10.1038/ng.2797 24097068 PMC3838666

[31] Lambert JC, Ibrahim-Verbaas CA, Harold D, Naj AC, Sims R, Bellenguez C, et al Meta-analysis of 74,046 individuals identifies 11 new susceptibility loci for Alzheimer’s disease. *Nature Genetics*, 2013, 45(12), 1452–1458. 10.1038/ng.2802 24162737 PMC3896259

[32] Shen L, Thompson PM, Potkin SG, Bertram L, Farrer LA, Foroud TM, et al Genetic analysis of quantitative phenotypes in AD and MCI: Imaging, cognition and biomarkers. *Brain Imaging Behav*. 2014;8(2):183–207. 10.1007/s11682-013-9262-z 24092460 PMC3976843

[33] Berger RL. Likelihood ratio tests and intersection-union tests *Advances in statistical decision theory and applications*. 1997 Birkhäuser Boston, 225–237. 10.1007/978-1-4612-2308-5_15

[34] Sen PK. Union–Intersection principle and constrained statistical inference. *Journal of Statistical Planning and Inference*. 2007 11 1;137(11): 3741–3752. 10.1016/j.jspi.2007.03.046

[35] Baselmans BML, Jansen R, Ip HF, van Dongen J, Abdellaoui A, van de Weijer MP, et al Multivariate genome-wide analyses of the well-being spectrum. *Nat Genet*. 2019 3;51(3):445–451. 10.1038/s41588-018-0320-8 Epub 2019 Jan 14. 30643256

[36] Akaike HA. Bayesian extension of the minimum AIC procedure of autoregressive model fitting. *Biometrika*. 1979 8; 66 (2):237–242. 10.1093/biomet/66.2.237

[37] Giambartolomei C, Vukcevic D, Schadt EE, Franke L, Hingorani AD, Wallace C, et al Bayesian test for colocalisation between pairs of genetic association studies using summary statistics. PLoS Genet. 2014 5 15;10(5):e1004383 10.1371/journal.pgen.1004383 24830394 PMC4022491

[38] Schizophrenia Working Group of the Psychiatric Genomics Consortium. Biological insights from 108 schizophrenia-associated genetic loci. Nature. 2014 7 24;511(7510):421–7. 10.1038/nature13595 25056061 PMC4112379

[39] 1000 Genomes Project Consortium, Auton A, Brooks LD, Durbin RM, Garrison EP, Kang HM, et al A global reference for human genetic variation. Nature. 2015 10 1;526(7571):68–74. 10.1038/nature15393 26432245 PMC4750478

[40] Deng Y, Pan W. Improved Use of Small Reference Panels for Conditional and Joint Analysis with GWAS Summary Statistics. *Genetics*. 2018 6 1;209(2): 401–408. 10.1534/genetics.118.300813 29674520 PMC5972416

[41] Pruim RJ, Welch RP, Sanna S, Teslovich TM, Chines PS, Gliedt TP, et al LocusZoom: regional visualization of genome-wide association scan results. Bioinformatics. 2010 9 15;26(18):2336–7. 10.1093/bioinformatics/btq419 20634204 PMC2935401

[42] Wilson DJ. The harmonic mean p-value for combining dependent tests. *PNAS* 1 22, 2019 116 (4) 1195–1200; first published January 4, 2019. 10.1073/pnas.1814092116 30610179 PMC6347718

[43] Besag J, Clifford P. Sequential Monte Carlo p-values. Biometrika. 1991 6;78(2):301–304. 10.1093/biomet/78.2.301

