# Supplementary material for: A powerful and versatile colocalization test
Source: PLoS Comput Biol. 2020 Apr 10;16(4):e1007778. doi: 10.1371/journal.pcbi.1007778 (PMC7176287; doi:10.1371/journal.pcbi.1007778)
Supplement: S1 Text — (DOCX) [file pcbi.1007778.s001.docx]

Text S1. Supplementary for "A Powerful and Versatile Colocalization Test"

## S.1 Summary of Existing Methods

We give a brief review of some of the existing methods.

**JLIM** [1]

H_0_: No colocalization.

H_1_: Colocalization.

Assumption: At most one causal variant for each trait.

Data: 2 traits. The second trait needs individual level data for permutation use.

Suppose the Z statistics for the first and second traits are $\mathbf{Z}=(z_{1},...,z_{q})'$ and $\mathbf{W}=(w_{1},...,w_{q})'$ respectively. $\theta$ is a threshold for neighborhood, recommended as $\sqrt{0.8}$. If two SNPs' correlation is higher than $\theta$, they are within each other's LD neighborhood. Note that there are two ways to define the set of SNPs that are within LD neighborhood of a SNP $i$. The first way is to estimate LD by the reference panel, resulting in $\mathcal{N}_{\theta}^{1}(i)$. The second way is to estimate LD by the individual level data of the second trait, resulting in $\mathcal{N}_{\theta}^{2}(i)$. Suppose the largest element of $\mathbf{Z}$ is $z_{m}$. The test statistic is

$$\Lambda=\sum_{i\in\mathcal{N}_{\theta}^{1}(m)} e^{\frac{1}{2}(z_{i}^{2}-z_{m}^{2})}\times({w_{i}}^{2}-\max_{j\notin\mathcal{N}_{\theta}^{2}(i)} {w_{j}}^{2}).$$

The null distribution of $\Lambda$ is obtained by permuting the individual level data of the second trait.

**Coloc** [2]

Data: 2 traits (summary statistics).

H_0_: A shared common causal variant (colocalization).

Coloc tests whether the coefficients from two separate regressions may be considered proportional. Some people say the proportionality assumption is used to test whether the traits have the exactly same causal locations, which is different from our definition of colocalization (traits share at least one causal location, but not necessarily all the causal locations), and does not limit to one causal location per trait. However, it is easy to construct a situation where two traits share the exactly same two causal variants but the effects are not proportional. For instance, suppose SNP 1 and SNP 2 are in low LD (correlation = 0.1). SNP 1's effects on trait 1 and trait 2 are 1 and 0.1 respectively. SNP 2's effects on trait 1 and trait 2 are 0.1 and 1 respectively. In the regression models, SNP 1's effect on trait 1 is larger than its effect on trait 2, while SNP 2's effect on trait 1 is smaller than its effect on trait 2, which means the coefficients are not proportional, even though both SNPs have colocalization. Hence, it may be best to assume at most one shared causal location when using the proportionality assumption to test colocalization.

Suppose the estimated coefficients and their covariance matrix from the joint model trait 1 vs. SNPs are ${\hat{\mathbf{b}}}_{1}=(\hat{b}_{11},...,\hat{b}_{1q})'$ and ${\hat{\mathbf{V}}}_{1}$. The estimated coefficients and their covariance matrix from the joint model trait 2 vs. SNPs are ${\hat{\mathbf{b}}}_{2}=(\hat{b}_{21},...,\hat{b}_{2q})'$ and ${\hat{\mathbf{V}}}_{2}$. The test statistic is

$$\mathbf{u'}\mathbf{V}^{-1}\mathbf{u,}$$

where $\mathbf{u=}{\hat{\mathbf{b}}}_{1}-{\hat{\mathbf{b}}}_{2}/\eta$ and $\mathbf{V=}{\hat{\mathbf{V}}}_{1}+{\hat{\mathbf{V}}}_{2}/\eta^{2}$. $\eta$ is a parameter to be estimated. Under the null hypothesis of proportionality, $\mathbf{b}_{1}=\mathbf{b}_{2}/\eta$.

**Coloc.abf** (a Bayesian colocalization test) [3]

Data: 2 traits (summary statistics).

H_0_: No association with either trait.

H_1_: Association with trait 1 only.

H_2_: Association with trait 2 only.

H_3_: Association with two traits, two independent SNPs.

H_4_: Association with two traits, one shared SNP.

Assumption 1: The causal variant is included in the set of variants.

Assumption 2: There is at most one causal variant for each trait.

Coloc.abf gives the evidence of H_4_ in the form of posterior probability. [3] mentioned their method still worked even if assumption 2 was violated, because it focused on the strongest signals.

**HEIDI** [4]

Data: 2 traits (trait + gene expression; summary statistics).

H_0_: There is a single causal variant affecting both traits.

Like coloc, HEIDI's null hypothesis implies colocalization, and it tests this hypothesis by examining whether the effects are proportional.

Suppose we have the same ${\hat{\mathbf{b}}}_{1}$, ${\hat{\mathbf{V}}}_{1}$, ${\hat{\mathbf{b}}}_{2}$ and ${\hat{\mathbf{V}}}_{2}$ as in coloc. Denote $\hat{b}_{1k}/\hat{b}_{2k}$ by $\hat{b}_{xy(k)}$. Assume the SNP with the smallest p-value on trait 2 (gene expression) corresponds to $\hat{b}_{xy(\mathrm{top})}$. Define $\hat{d}_{k}=\hat{b}_{xy(k)}-\hat{b}_{xy(\mathrm{top})}$ and $z_{d(k)}=\hat{d}_{k}/\sqrt{\mathrm{var}(\hat{d}_{k})}$. The test statistic is

$$\sum_{k=1}^{q} {z_{d(k)}}^{2}.$$

According to [4], $\mathrm{var}(\hat{d}_{k})$ can be calculated and the distribution of the test statistic can be approximated by some method.

**eCAVIAR** [5]

Data: 2 traits (trait + gene expression; summary statistics).

H_0_: No colocalization.

H_1_: Colocalization.

eCAVIAR computes the posterior probability of colocalization and is robust to the presence of allelic heterogeneity (AH), which means there is more than one causal variant for a trait. [5] showed eCAVIAR performs better than coloc when AH exists. Although eCAVIAR allows multiple causal variants, since it looks at all the possible situations, it still has to assume a limited maximum number of causal SNPs (e.g. 6), or it may be computationally infeasible.

## S.2 Obtaining the Weights for Model Averaging

We obtain the weights following the idea in [6]. Denote the observed Z-scores by $\mathbf{Z=(}Z_{jk}\mathbf{)}$. For $\triangle_{t}$, we assume $vec(\mathbf{Z})\sim MVN(vec(\mathbf{Z}^{\boldsymbol{(}t)}),\mathbf{C})$, where $\mathbf{C}$ is estimated as $\boldsymbol{R}_{1}\otimes\boldsymbol{R}_{2}$. $\mathbf{Z}^{\boldsymbol{(}t)}\boldsymbol{=}\mathbf{(}Z_{jk}^{(t)}\mathbf{)}$ contains the parameters to be estimated under

$$\left\{ \begin{aligned} Z_{jk}^{(t)}\neq0 ((j,k)\in\triangle) \\ Z_{jk}^{(t)}=0 ((j,k)\notin\triangle) \end{aligned} \right..$$

Since there is only one data point $vec(\mathbf{Z})$, we can simply estimate ${\hat{\mathbf{Z}}}^{\boldsymbol{(}t)}\boldsymbol{=}\mathbf{(}\hat{Z}_{jk}^{(t)}\mathbf{)}$ using

$$\hat{Z}_{jk}^{(t)}=\left\{ \begin{aligned} Z_{jk} ((j,k)\in\triangle) \\ 0 ((j,k)\notin\triangle) \end{aligned} \right.,$$

which leads to log-likelihood

$$l=-\frac{1}{2}[log(||\mathbf{C}||)+(vec(\mathbf{Z})-vec({\hat{\mathbf{Z}}}^{\boldsymbol{(}t)}))'\mathbf{C}^{-1}(vec(\mathbf{Z})-vec({\hat{\mathbf{Z}}}^{\boldsymbol{(}t)}))+pqlog(2\pi)].$$

Denote the size of $\triangle_{t}$ by $\left| \triangle_{t} \right|$. $\left| \triangle_{t} \right|$ is also the number of nonzero parameters to be estimated in $\mathbf{Z}^{\boldsymbol{(}t)}$. Then the corresponding AICc is

$${AICc}_{t}=2\left| \triangle_{t} \right|+\frac{2\left| \triangle_{t} \right|(\left| \triangle_{t} \right|+1)}{1-\left| \triangle_{t} \right|-1}-2l=-2-2l.$$

The weight for $\triangle_{t}$ is

$$w_{t}=e^{-\frac{1}{2}{AICc}_{t}}.$$

## S.3 Selecting the Tuning Parameter $\boldsymbol{\theta}$ for CMC

Some results for different $\theta$'s (0.05, 0.1 and 0.2) are shown in the main article (Table 1). We did more simulation studies in scenarios closer to real situations to examine the effect of $\theta$. The settings were the same as what we had for Table 4 in the main article. As Table S1 shows, smaller $\theta$'s led to higher rejection rates. $\theta=0.05$ and $\theta=0.1$ were able to control type I errors while maintaining decent power. To further examine how $\theta$ affects type I errors in more extreme scenarios (where the effect sizes are much larger), we did simulation studies of regions A1 and A2 with increased true effect sizes. We multiplied the effect sizes by a constant $o$ and plotted the type I error rates in Fig. 1 and Fig. 2. When the effect sizes became much larger, $\theta=0.05$ and $\theta=0.1$ still had decent type I error rates. Note that sometimes the type I error rate even went down as the effect sizes went up. This is probably because when the nonzero effects are larger, they are more likely to be diagnosed as nonzero by comparing observed Z-scores with the selected threshold, and thus the simulated test statistics may tend to be slightly larger since more Z-scores have nonzero means. Even though $\theta=0.05$ seems to be the best choice based on these results, considering the variety of situations in reality, it may be safer to use $\theta=0.1$, which is slightly more conservative than $\theta=0.5$. Hence, we recommend using $\theta=0.1$ by default.

**Table S1.** Rejection rates for CMC with different $\theta$'s. 1000 iterations. $\alpha=0.05$. Same subjects for two traits. $B={10}^{3}$. The settings for regions A1-A2 and B1-B3 were the same as in Table 4 (main text). Regions A1-A2: without colocalization (type I errors). Regions B1-B3: with colocalization (power).

| Region | # SNPs  (*) | $\theta$ | | | | |
| --- | --- | --- | --- | --- | --- | --- |
|  |  | 0.02 | 0.05 | 0.1 | 0.2 | 0.3 |
| A1 | 43 (2/0/0) | 0.036 | 0.024 | 0.014 | 0.010 | 0.005 |
| A2 | 58 (17/2/0) | 0.053 | 0.041 | 0.030 | 0.024 | 0.021 |
| B1 | 28 (9/4/1) | 0.312 | 0.291 | 0.276 | 0.248 | 0.230 |
| B2 | 53 (7/18/2) | 0.594 | 0.575 | 0.542 | 0.515 | 0.495 |
| B3 | 62 (12/6/2) | 0.784 | 0.761 | 0.721 | 0.674 | 0.645 |

**Fig. 1.** Type I errors for CMC with different $\theta$'s and true effect sizes multiplied by $o$. Region A1.

**Fig. 2.** Type I errors for CMC with different $\theta$'s and true effect sizes multiplied by $o$. Region A2.

## S.4 Additional Explanations for Table 1

JLIM had very low rejection rates when the causal SNP for trait 2 was not SNP 1. Note that none of the correlations was larger than 0.89, which was the threshold for neighborhoods adopted in JLIM. Hence, JLIM only considered SNP 1 as causal for trait 1 as there was no other SNP in the neighborhood. As a result, the test statistic became

$$\Lambda={w_{1}}^{2}-\max_{j\neq1} {w_{j}}^{2},$$

where $w_{j}$ was the Z statistic for trait 2- SNP $j$ association. The definition of the test statistic $\Lambda$ can be found in the supplementary materials.

(1) If the causal SNP for trait 2 was SNP 3, ${w_{3}}^{2}$ would be much larger than ${w_{1}}^{2}$, so $\Lambda$ would be very small.

(2) If there was no causal SNP for trait 2 or the data was permuted, the difference between ${w_{1}}^{2}$ and $\max_{j\neq1} {w_{j}}^{2}$ would be much smaller than that in the previous case.

As a result, JLIM did not reject the null, since none of $\Lambda$ from permutations was smaller than $\Lambda$ from the original data. In conclusion, the difference between JLIM and the conditional method with permutations in terms of type I error was because the test statistics were constructed differently. $\Lambda$ was less significant in case (1) than in (2), while $P$ was more significant in case (1) than (2).

Besides, notice that when the coefficients for the first trait were not changed, the p-value for testing the first SNP was not changed. Since we used the true X'X (i.e. individual-level data) to build the conditional model for trait 2, the coefficient and the standard error for the first SNP only depended on the first coefficient and the random seed (i.e. the error term):

$$\tilde{\boldsymbol{\beta}}\boldsymbol{=}\mathbf{(X'X)}^{\boldsymbol{-1}}\mathbf{X}'\mathbf{Y}\mathbf{＝}\mathbf{(X'X)}^{\boldsymbol{-1}}\mathbf{X}'(\boldsymbol{X\beta+\varepsilon})=\boldsymbol{\beta+}\mathbf{(X'X)}^{\boldsymbol{-1}}\mathbf{X}'\boldsymbol{\varepsilon,}$$

$$\tilde{\sigma}^{2}=\frac{\mathbf{Y}^{'}\mathbf{Y}-\mathbf{Y}^{\mathbf{'}}\mathbf{X}\left( \mathbf{X}^{\mathbf{'}}\mathbf{X} \right)^{\boldsymbol{-1}}\mathbf{X}^{'}\mathbf{Y}}{n-q}$$

$$=\frac{1}{n-q}[\boldsymbol{\beta'X'X\beta+2\beta'X'\varepsilon+\varepsilon'\varepsilon-\beta'X'X\beta-2\beta'X'\varepsilon-\varepsilon'X}\mathbf{(X'X)}^{\boldsymbol{-1}}\mathbf{X}'\boldsymbol{\varepsilon}]=\frac{\boldsymbol{\varepsilon'}(\mathbf{I-X}\mathbf{(X'X)}^{\boldsymbol{-1}}\mathbf{X}')\boldsymbol{\varepsilon}}{n-q},$$

$$\mathrm{Cov}\boldsymbol{(}\tilde{\boldsymbol{\beta}}\boldsymbol{)=}\tilde{\sigma}^{2}\mathbf{(X'X)}^{\boldsymbol{-1}}\boldsymbol{=}\frac{\boldsymbol{\varepsilon'}(\mathbf{I-X}\mathbf{(X'X)}^{\boldsymbol{-1}}\mathbf{X}')\boldsymbol{\varepsilon}\mathbf{(X'X)}^{\boldsymbol{-1}}}{n-q}.$$

Hence, even if we changed $\beta_{3}$ or $\beta_{4}$, it would not affect the inference on $\beta_{1}$, as long as the random seed was fixed.

## S.5 Plots of Effect Sizes for Table 4

**Fig. 3.** Effect sizes in Region A1.


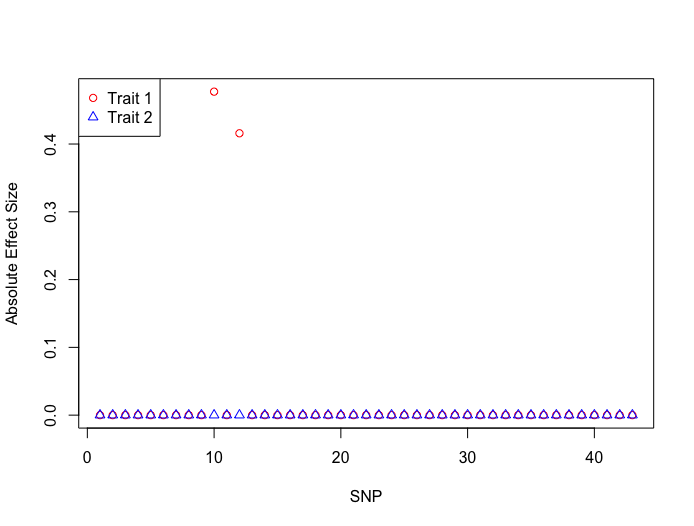


**Fig. 4.** Effect sizes in Region A2.


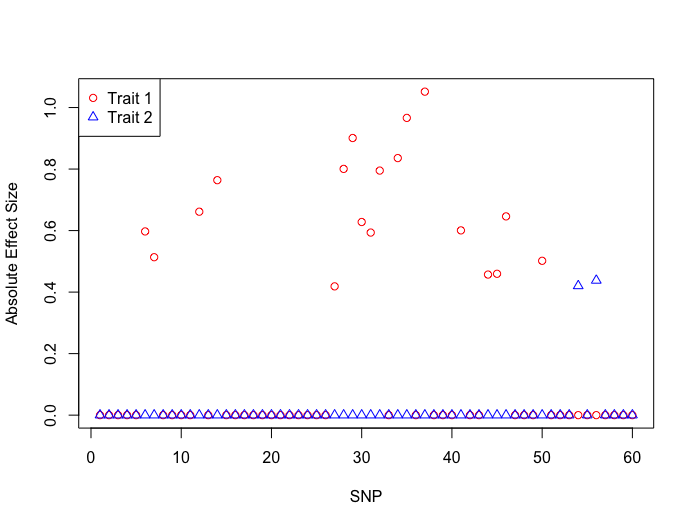


**Fig. 5.** Effect sizes in Region B1.


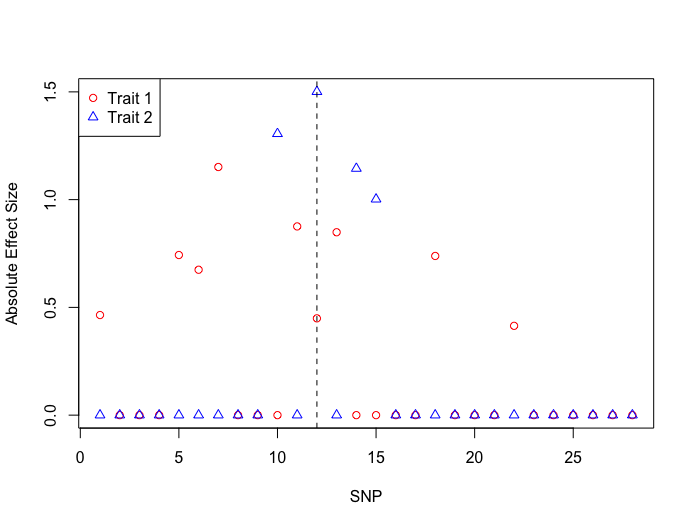


**Fig. 6.** Effect sizes in Region B2.


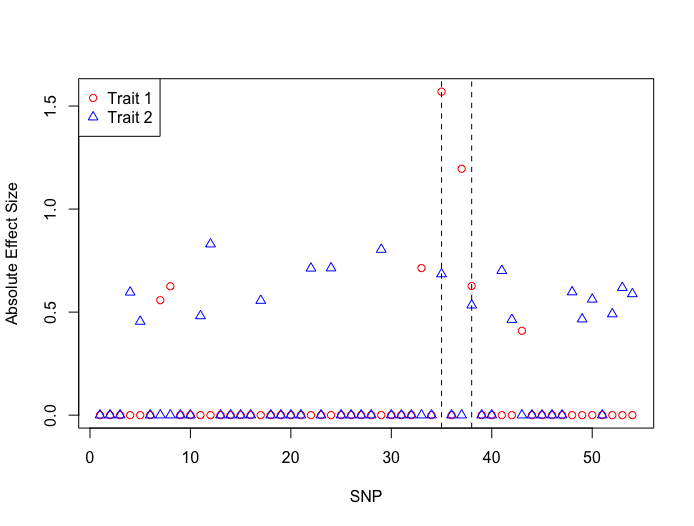


**Fig. 7.** Effect sizes in Region B3.


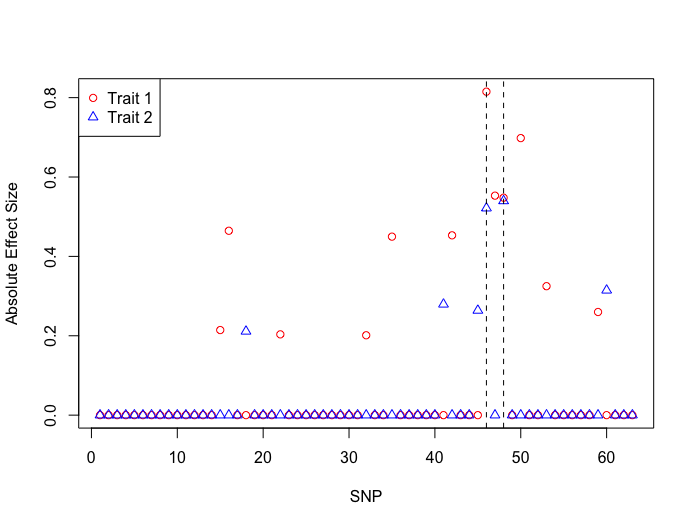


**Fig. 8.** Effect sizes in Region B4.


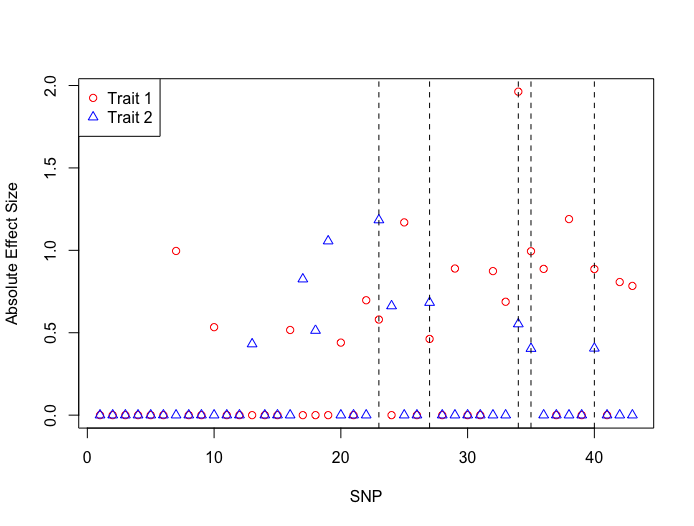


**Fig. 9.** Effect sizes in Region B5.


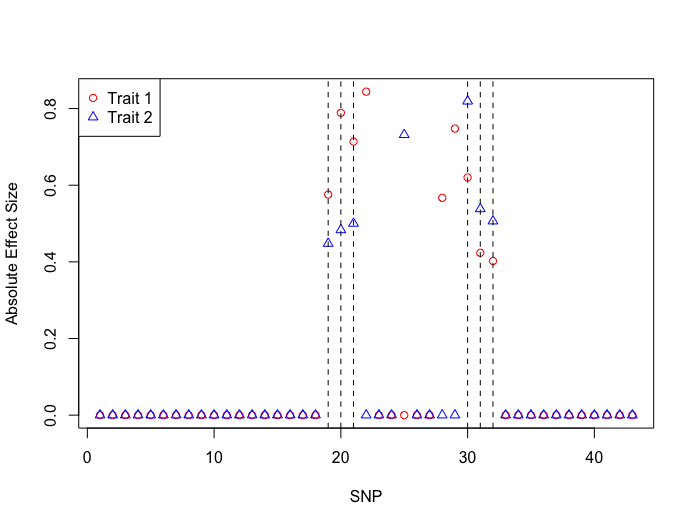


## S.6 Results for IGAP and ADNI Data

We applied the methods to the largest AD GWAS data by IGAP (International Genomics of Alzheimer's Project) [7], along with the ADNI (Alzheimer's Disease Neuroimaging Initiative) data [8], only looking at the chromosomes that had at least one SNP associated with AD in the IGAP data (p-value < 5e-8). There were 711 subjects in ADNI with their genotype, expression and covariate data available. First, we found all the SNPs in the ADNI data that were marginally significant for AD. Then we combined them into loci by the same approach as used for the lipid data, considering the LD structure of SNPs and their marginal p-values for AD. For each locus, we tested each gene’s expression level against each SNP in the locus to determine which genes should be considered in the analyses. If a gene’s expression turned out to be significantly associated with at least one of the SNPs in the locus (p-value < 5e-8), we would test colocalization of AD and this gene’s expression in this locus. We also applied eCAVIAR [5], which gave a colocalization posterior probability (CLPP) for each SNP. We took the maximum of the SNPs' CLPPs in each locus and compared it with a cutoffs.

The results of different methods are shown in Table S2. A locus was found to have colocalization as long as a colocalization happened between AD and expression of any genes in the locus. At the significance level 5e-8, the conditional method with the Bonferroni adjustment and CMC gave the same results, detecting fewer loci with colocalization than eCAVIAR for the given cut-offs, but note that, since the cut-offs for the two methods were different, their results were not directly comparable. Also, with a loose cutoff, CMC had more discoveries than CB and the number of significant loci matched eCAVIAR's. The coloc method seemed to find a lot more loci with colocalization, especially on chromosome 19, but again likely because there was not enough evidence to reject its null hypothesis (colocalization).

**Table S2.** Numbers of loci with colocalization. The numbers of SNPs for marginal only considered those within the analyzed loci, while the cutoff for testing each SNP or lead SNP was $\alpha=0.05/\#SNPs in that chromosome$. For the methods testing each locus, $\alpha=0.05/\#\mathrm{loci}$ (or 5e-8). The cutoff for eCAVIAR was 0.1 (0.8).

| Chr | Marginal | | Regional | | | | | | | | |
| --- | --- | --- | --- | --- | --- | --- | --- | --- | --- | --- | --- |
|  | # SNPs | # Significant SNPs | # Loci | # Loci with Colocalization | | | | | | | # Significant Lead SNPs* |
|  |  |  |  | JLIM | CB | CMC | MA | Coloc | HEIDI** | eCAVIAR |  |
| 2 | 4 | 1 (1) | 1 | 0 (0) | 0 (0) | 0 (0) | 0 (0) | 0 (0) | 0 (1) | 0 (0) | 0/0 |
| 6 | 19 | 1 (1) | 2 | 0 (0) | 1 (0) | 1 (0) | 1 (0) | 1 (1) | 1 (1) | 1 (1) | 0/1 |
| 8 | 7 | 1 (1) | 2 | 1 (1) | 0 (0) | 1 (0) | 1 (0) | 2 (2) | 1 (1) | 1 (1) | 1/1 |
| 11 | 22 | 2 (1) | 3 | 0 (0) | 0 (0) | 1 (0) | 1 (0) | 3 (3) | 2 (2) | 1 (0) | 1/1 |
| 18 | 3 | 1 (1) | 1 | 1 (1) | 1 (1) | 1 (1) | 1 (1) | 0 (0) | 0 (1) | 1 (1) | 1/1 |
| 19 | 107 | 1 (0) | 24 | 2 (2) | 0 (0) | 0 (0) | 0 (0) | 23 (24) | 1 (1) | 0 (0) | 0/3 |

We used some LocusZoom plots [9] to show the difference among the methods in different regions. In Fig. 10, the situation is the opposite to that in Fig 3 in the main text. The SNPs were significant in the marginal analysis but not in the conditional analysis, so JLIM yielded a significant result while the conditional method did not.

Fig. 11 and Fig. 12 show the difference between JLIM, the conditional method and coloc. Note that the null hypothesis of JLIM and the conditional method is that there is no colocalization, while the null hypothesis of coloc is that the effect sizes in the joint models for the two traits are proportional, suggesting colocalization. In Fig. 11, SNP rs11218343 seemed to be significant for both traits, which led both JLIM and the conditional method to conclude colocalization. In Fig. 12, neither JLIM nor the conditional method detected colocalization, because the effects on trait 2 were not significant enough. Fig. 13 shows the effect sizes in these two cases. Method coloc seemed to suggest the effects were more likely to be proportional in the latter case (Fig. 13, right) than in the former case (Fig. 13, left) somehow, leading to its different conclusions from that of JLIM and the conditional method.

**Fig. 10.** One locus associated with schizophrenia on chromosome 8. JLIM detected colocalization while the conditional method did not. Top: LocusZoom plots of 6 SNPs' p-values in marginal analysis. Bottom: LocusZoom plots of the same SNPs' p-values in conditional analysis.

**Fig. 11.** One locus associated with schizophrenia on chromosome 18. JLIM and the conditional method detected colocalization, while coloc did not. Top: LocusZoom plots of 3 SNPs' p-values in marginal analysis. Bottom: LocusZoom plots of the same SNPs' p-values in conditional analysis. Smaller p-values are truncated at 1e-15.

**Fig. 12.** One locus associated with schizophrenia on chromosome 11. JLIM and the conditional method did not detect colocalization, while coloc did. Top: LocusZoom plots of 6 SNPs' p-values in marginal analysis. Bottom: LocusZoom plots of the same SNPs' p-values in conditional analysis. Smaller p-values are truncated at 1e-15.

**Fig. 13.** Effect sizes of the SNPs in the conditional models. Left: one locus with 3 SNPs on chromosome 18, corresponding to Fig. 4. Right: one locus with 6 SNPs on chromosome 11, corresponding to Fig. 5. Lines: regression lines.


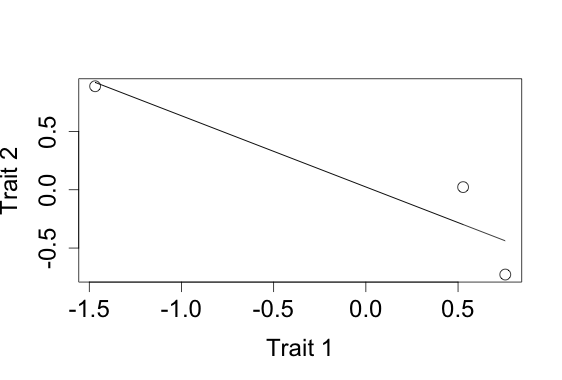

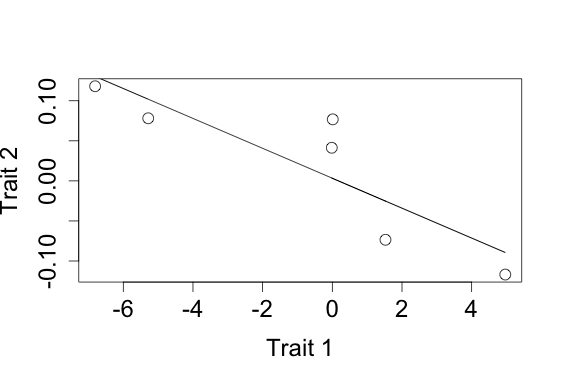


## References

1. Chun S, Casparino A, Patsopoulos NA, Croteau-Chonka DC, Raby BA, De Jager PL, et al. Limited statistical evidence for shared genetic effects of eQTLs and autoimmune-disease-associated loci in three major immune-cell types. Nat Genet. 2017 Apr;49(4):600-605. doi: 10.1038/ng.3795.

2. Wallace C, Rotival M, Cooper JD, Rice CM, Yang JH, McNeill M, et al. Statistical colocalization of monocyte gene expression and genetic risk variants for type 1 diabetes. Hum Mol Genet. 2012 Jun 15;21(12):2815-24. doi: 10.1093/hmg/dds098.

3. Giambartolomei C, Vukcevic D, Schadt EE, Franke L, Hingorani AD, Wallace C, et al. Bayesian test for colocalisation between pairs of genetic association studies using summary statistics. PLoS Genet. 2014 May 15;10(5):e1004383. doi: 10.1371/journal.pgen.1004383.

4. Zhu Z, Zhang F, Hu H, Bakshi A, Robinson MR, Powell JE, et al. Integration of summary data from GWAS and eQTL studies predicts complex trait gene targets. *Nature Genetics*, 2016 May; *48*(5), 481-7. doi: 10.1038/ng.3538.

5. Hormozdiari F, van de Bunt M, Segrè AV, Li X, Joo JWJ, Bilow M, et al. Colocalization of GWAS and eQTL Signals Detects Target Genes. Am J Hum Genet. 2016 Dec 1;99(6):1245-1260. doi: 10.1016/j.ajhg.2016.10.003.

6. Baselmans BML, Jansen R, Ip HF, van Dongen J, Abdellaoui A, van de Weijer MP, et al. *Nat Genet.* 2019 Mar; 51(3):445-451. doi: 10.1038/s41588-018-0320-8. Epub 2019 Jan 14.

7. Lambert JC, Ibrahim-Verbaas CA, Harold D, Naj AC, Sims R, Bellenguez C, et al. Meta-analysis of 74,046 individuals identifies 11 new susceptibility loci for Alzheimer’s disease. *Nature Genetics*, 2013, *45*(12), 1452–1458. http://doi.org/10.1038/ng.2802.

8. Shen L, Thompson PM, Potkin SG, Bertram L, Farrer LA, Foroud TM, et al. Genetic analysis of quantitative phenotypes in AD and MCI: Imaging, cognition and biomarkers. *Brain Imaging and Behavior*, 8, 183-207 (2014).

9. Pruim RJ, Welch RP, Sanna S, Teslovich TM, Chines PS, Gliedt TP, et al. LocusZoom: regional visualization of genome-wide association scan results. Bioinformatics. 2010 Sep 15;26(18):2336-7. doi: 10.1093/bioinformatics/btq419.
